# Understanding implementation determinants of universal school meals through an equity-driven mixed methods approach

**DOI:** 10.1186/s43058-025-00713-0

**Published:** 2025-04-15

**Authors:** Gabriella M. McLoughlin, Molly Kerstetter, Yerusalem Yohannes, Omar Martinez, Resa M. Jones, Ross C. Brownson, Jennifer O. Fisher

**Affiliations:** 1 Department of Social and Behavioral Sciences, Temple University College of Public Health, Philadelphia, PA USA; 2 College of Medicine, University of Central Florida, Orlando, FL USA; 3 Department of Epidemiology and Biostatistics, Temple University College of Public Health, Philadelphia, PA USA; 4 Fox Chase Cancer Center, Temple University Health, Philadelphia, PA USA; 5 Siteman Cancer Center, Washington University School of Medicine, St. Louis, MO USA; 6 Brown School of Social Work, Washington University in St. Louis, St. Louis, MO USA

**Keywords:** Implementation science, Health equity, Implementation Mapping, Policy, Food security

## Abstract

**Background:**

Policies, such as Universal School Meals (USM), are essential for preventing inequities in chronic disease risk among socially and economically marginalized populations. Implementing USM reduces food insecurity and obesity risk, among other academic/health outcomes; unfortunately, across the nation student participation (i.e., reach) is lower than expected, limiting its public health impact. Grounded in implementation science and health equity frameworks, this study aimed to: 1) investigate the determinants of implementing USM in a large, urban school district and 2) assess key challenges and supports across schools with varying levels of participation in USM.

**Methods:**

A needs and assets assessment was undertaken in the 2023–2024 academic year with the School District of Philadelphia to address implementation-related challenges for USM as part of a broader Implementation Mapping process. Overall, 8 schools (6 middle; 2 high) participated in a convergent mixed methods study comprising qualitative interviews, surveys, and mealtime observations. Data collection was grounded in the Consolidated Framework for Implementation Research (CFIR) and Health Equity Measurement Framework. Interviews were deductively coded through the CFIR; barriers were coded negatively (either -1 or -2), supports coded positively (+ 1 or + 2), and neutral determinants coded as 0. Schools were grouped into low, moderate, and high meal participation for disaggregated analysis and comparison of determinants across reach.

**Results:**

193 participants included teachers (29%), parents (26%), students (middle 14%; high school 10%), administrators (13.5%), and food service personnel (11%). Participants identified as Black/African American (43%), White (26%), Hispanic/Latino (20%), Asian (5%), Middle Eastern (1.8%), and other (3.8%). The strongest facilitators of USM implementation were Mid-level Leaders (i.e., climate leaders; M = 1.29[-1,2]) and High-level Leaders (i.e., administrators; M = 0.96[-1,2]); strongest negative USM determinants were Market Pressure (i.e., competitive foods; M = -1.35[-2,0]), and Relative Priority (M = -1.17[-2,-1]). Emerging differences between low and moderate/high participation groups were found in Culture, Assessing Needs of Recipients, Access to Knowledge/Information, Human Equality-Centeredness, and Implementation Leads. Overall, higher participation schools reported less stigma, more equitable implementation procedures, and more involvement from food service managers than lower participation schools.

**Conclusions:**

Equity-focused strategies targeting key issues within and outside the school setting are needed to reduce stigma and increase capacity for implementation.

**Supplementary Information:**

The online version contains supplementary material available at 10.1186/s43058-025-00713-0.

Contributions to the literature

- This study documents the process and findings from a community-engaged needs assessment, which will lead to the development of implementation strategies to enhance USM implementation and advance the field of policy implementation science.
- We worked collaboratively with a Community Advisory Board who provided invaluable feedback; other researchers can use our process as a guide for collaborating with community members.
- The convergent mixed methods approach facilitated understanding of determinants across a range of reach/participation levels, which allows us to develop tailored implementation strategies.
- Methods can be applied within the US and globally given increased attention toward USM

## Background

Overweight and obesity is a major risk factor for preventable chronic health conditions such as cardiovascular disease [1, 2]. Currently in the United States (US) over 19% of children ages 2–19 have obesity; inequities exist between white (16%), non-Latinx Black (24%), and Latino youth (25%) [3]. Recent evidence suggests inequities in obesity have increased since the COVID-19 pandemic, especially for adolescents within the US [4] and globally among low- and middle-income countries [5]. Given the complex community and population-level factors that influence health outcomes (i.e., poverty, discrimination, inadequate access to healthy food) [6], policy, systems, and environmental (PSE) approaches are necessary to mitigate obesity risk and achieve equitable outcomes for socially and economically marginalized populations [7–9].

Research indicates that providing healthy school meals is associated with higher quality nutritional intake and reduced obesity prevalence, especially in low-income students [9–12]. Thus, increasing access to healthy school meals is a critical step to mitigating inequities in obesity prevalence in youth [11]. Universal School Meals (USM) is an important policy provision [13], embedded within the National School Lunch Program, where all students in high-poverty schools serving more than 25% low-income students can receive free school breakfast and lunch. USM adoption is also associated with quality of dietary intake, food security, and academic achievement outcomes observed through randomized trials and longitudinal studies [14–16]. Recent evidence from a state-wide longitudinal study in California demonstrated that schools participating in USM were associated with a 0.60-percentage-point net decrease in obesity prevalence after policy adoption (95% confidence interval: − 1.07 to − 0.14 percentage points, P = 0.01) compared with eligible, nonparticipating schools. This equated to a 2.4% relative reduction when accounting for baseline prevalence [17]. Therefore, USM is a key PSE approach for equitable obesity prevention. Although the research is more limited in low- and middle-income countries, several organizations, including the World Food Programme, are building evidence and capacity to make the case for USM globally [18, 19].

Despite many benefits associated with USM, schools cite financial challenges for implementation and lack of uptake among students [13]. Reports highlight consistent increases in adoption among eligible schools and districts over the last 5 years [13]; in the 2022 to 2023 school year, 82% of eligible schools had implemented CEP, providing 19.9 million children access to UFSMs [20]. To advance this provision and based on promising findings, 9 states (California, Colorado, Maine, Massachusetts, Michigan, Minnesota, New Mexico, Nevada, and Vermont) moved to a state-wide model in 2023–2024. Despite advancements in state and local adoption, student participation (i.e., reach) in USM remains low; only 30–40% of students partake in breakfast and 50–60% in lunch, from a statewide study New York [21] and in the School District of Philadelphia (SDP) [22, 23]. Students can participate at two time points during the school day (breakfast and lunch); therefore, participation varies among students and over the school year. Programs and policies designed to mitigate health inequities for obesity cannot make the most impact if they are not reaching their target population. Thus, optimizing reach of USM will enhance its impact on addressing inequities in child obesity.

Dissemination and implementation science facilitates the process by which evidence-based interventions are implemented and sustained in practice [24]. This is achieved by developing implementation strategies designed to enhance implementation of evidence-based interventions [25]. Such strategies can be chosen through a variety of ways, but Implementation Mapping is a key method to ensure a participant-driven process [26]. Although implementation science provides systematic approaches for increasing real-world impact of obesity prevention, health equity has not been a priority until recently [27–30], including the Consolidated Framework for Implementation Research (CFIR version 2) [31, 32]. By meaningfully integrating the work of health equity and social justice scholars into implementation science, we can anticipate and prevent implementation that causes further harm to socially and economically marginalized populations because their voices are central to the implementation process [33, 34]. Accordingly, leveraging implementation strategies to improve USM implementation is critical for equitable access to healthy school meals [35]. Finally, perspectives of students and families are not well represented in the literature [36, 37]; identifying policy recipient needs and implementation context is therefore essential to addressing obesity inequities [38, 39].

The CFIR, a commonly used determinants framework within implementation science, encompasses empirically-derived domains known to influence implementation of interventions including educational interventions [40]. Specific emphasis is placed on characteristics of the intervention (i.e., USM), inner context (i.e., school-level), outer context (i.e., school district, local/state/federal policy), characteristics of individuals (i.e., staff/provider) and implementation process (i.e., planning, engaging stakeholders). Further, the Health Equity Measurement Framework (HEMF) conceptually links together key CFIR domains such as socioeconomic, cultural, and political context, health policy context, material and social circumstances, with health resource utilization [41]. The framework makes key linkages between social determinants of health factors, the “need” for resources, utilization of health-promoting resources, and health outcomes, which harmonizes with the goals to improve USM implementation to maximizing student health outcomes. Following guidance by framework authors [27, 41], this blend will provide a comprehensive understanding of USM implementation determinants.

**Fig. 1:**
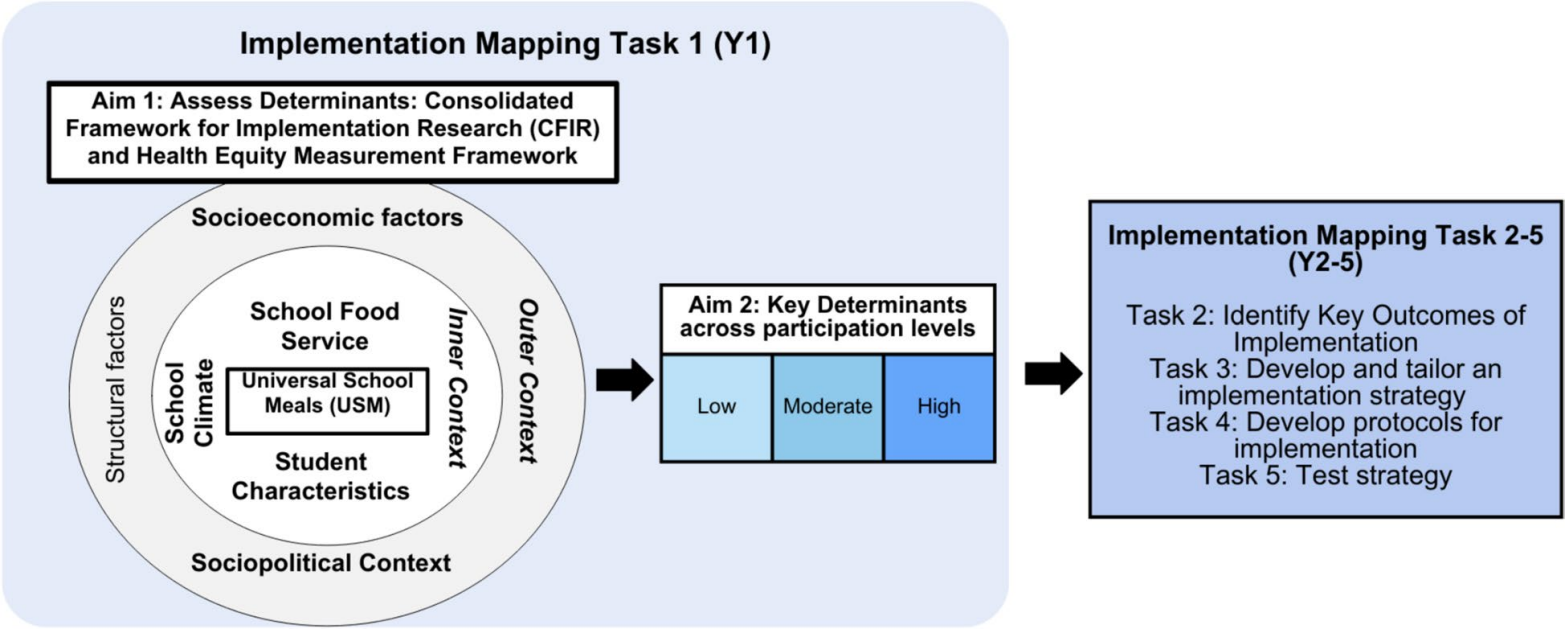
Study aims and alignment with implementation mapping

Grounded in participatory research, Intervention Mapping is a systematic process that relies on evidence, theory, and input from key stakeholders to guide intervention development [42]. Implementation Mapping comprises the same procedure, but with a focus on developing implementation strategies [26, 43] to enhance ongoing implementation efforts of an intervention. This process comprises five key tasks: 1) Needs and Assets Assessment; 2) Identify Outcomes for Implementation; 3) Develop and Tailor Implementation Strategies; 4) Develop Implementation Protocols; and 5) Evaluate Outcomes of the Strategy. This study reports the methods and results of Step 1 – Needs and Assets Assessment – which is part of an ongoing National Institutes of Health (NIH)-funded project (K01 HL166957-01, principal investigator [PI] GMM) in collaboration with the SDP [8, 31, 32] that will complete all five key tasks across the 5-year study. Figure 1 shows the conceptual overview and how this paper accomplishes a needs and assets assessment (Task 1), and how this will provide the foundation for the remaining tasks in Years 2–5 (Y2-5) of the Implementation Mapping study.

Given the overarching goal of increasing reach (i.e., participation in school meals) through Implementation Mapping, we sought to understand the key determinants to implementation and participation for schools adopting USM and to understand how these may differ across levels of participation to provide more in-depth information about how best to support schools in future years of the study. Accordingly, the two aims of this study were: 1) To investigate the determinants of implementing USM grounded in implementation science and health equity frameworks and 2) To assess key challenges and supports across schools with varying levels of participation in USM.

## Methods

This study employs a convergent mixed methods (QUAL- quant) design [44] to conduct a needs and assets assessment of USM implementation across the SDP.

### Setting and context

#### Partnership with the School District of Philadelphia

The SDP is the largest school district in Pennsylvania serving nearly 200,000 students; 50% of whom identify as Black/African American, 24% Latino, 14% white, 7% Asian, and 5% multiracial/other. All SDP schools provide breakfast and lunch at no cost to students because > 40% are from low-income households [13, 45]. In 2021 the principal investigator (GMM) began a partnership with SDP to collaborate on important aspects of school policy and to provide no-cost evaluation support for school nutrition programs. This led to meaningful collaboration on the evaluation of the SDP breakfast program [46] in addition to GMM serving on multiple committees for the school district; SDP collaborators provided substantial input on the NIH grant proposal funding the current study.

#### Community advisory board

As part of the broader NIH-funded project, we recruited and retained a Community Advisory Board (CAB) comprising individuals (N = 7) from academia (n = 1), non-profit organizations (n = 2), the Philadelphia department of public health (n = 1), former teachers (n = 1), parents (n = 1), and students in high schools (n = 2). The overarching purpose of the CAB is to act as a sounding board for the 5-year study; members were intentionally recruited before the needs assessment began so that they could provide input on school and participant recruitment materials, data collection approaches, and assist with interpretation of (blinded) data.

## Recruitment

There are a variety of roles and ways these roles influence USM implementation such as food service staff and managers (food preparation, service), classroom teachers (classroom feeding, influencers), school administrators (supporting staff, setting schedules), custodial/support staff (health and safety, implementation support), students (recipients, peer influencers), and parents (recipients, opinion leaders). Accordingly, we felt it important to recruit individuals from each of these participant groups from each school we worked with. Following guidelines from experts in health equity for best practices in recruitment [47], we took several steps to recruit and retain participants. Collaborating with the SDP office of research and evaluation, targeted sampling was used to choose schools from all major regions of the city of Philadelphia, with varying meal participation, and varying building sizes. The PI (GMM) contacted building principals to provide information about the study and goals for building capacity for implementation and invited them to participate in a video call to discuss the study further. Introduction calls were held during August and September 2023, and once schools agreed to participate, the research team visited schools to speak with staff and students to inform them about the study. This comprised multiple formats based on individual school needs such as presentations to staff during professional development days, meeting individually with food service staff and teachers before entering classrooms, and/or classroom presentations during brief pauses in instruction (for students). The team brought flyers in English and Spanish to display in classrooms and hallways (see Additional File 1), which provided QR codes to a REDCap consent (and assent) form to streamline recruitment. We also printed consent and assent forms (English and Spanish) based on schools’ requests and handed them out during school drop-offs and collection. To incentivize participation, all participants were given a $10 gift card for survey completion and a $15 gift card for participating in an interview with the study team (e-Amazon or Visa®).

## Data collection

### Research team

Interviews were led by the female (she/her pronouns) PI (GMM) who has extensive qualitative research experience and > 10 years working in school environments and leading school-based research. The PI trained four students (i.e., two master’s-level [MK, YY], two undergraduate) to conduct interviews. Training included reviewing draft interview guides, practicing and conducting mock interviews, and shadowing the study lead in initial rounds of interviews. Mentors and colleagues (JOF, RCB, OM, RMJ) provided critical oversight into the data collection and analysis procedures.

### Data collection instruments

#### Interview guides

The team developed interview guides grounded in the CFIR and HEMF for a range of study participant types: students, parents, teachers/staff, food service staff and managers, and administrators (i.e., principals, deans of students) (see Additional File 2). Questions targeted factors within the following CFIR domains: Innovation Characteristics (i.e., “How would you describe the healthiness of the meals currently served at school?” – Students); the Outer Setting (“Can you tell us about some of the city/district policies that may influence how school meals are served?”- Teachers/Staff); Inner Setting (“How would you say the culture of school meals is within your school?” – Food service); Characteristics of Individuals (“What common comments do you hear about breakfast and lunch service from students?” – Administrators); and Implementation Process (“What are some challenges about preparing and delivering breakfast and lunch?” – Food Service). HEMF-guided questions were integrated, for example in the Administrator interview guide in Individual Characteristics domain, we asked, “To what degree are community members aware of/engaged in conversations about school meals?” to align with the HEMF sociopolitical context. In the Inner Setting Domain, we asked Parents “Have you noticed any stigma, peer pressure or judgment related to eating school meals at your child’s school?” to align with the student characteristics/need domains of the HEMF. Interview guides were refined based on initial data collection experiences and reflection from the research team and feedback from the CAB during Fall 2023 meetings.

Except for some interviews with parents and staff members due to schedule preference, all interviews and observations took place at each school site during scheduled breaks (i.e., teacher prep periods), specific periods allowed for student interviews, or before/after school. Student interviews took place as focus groups with 2–4 participants in each conversation. All interviews were recorded and transcribed verbatim for analysis.

#### Field notes

To enhance data collection, extensive field notes were taken after each day of data collection to summarize high-level issues that arose. Field notes were also used to capture informal and impromptu conversations that occurred at schools with other personnel such as other administrators, custodial staff, and students, who did not participate in a formal interview. These notes and reflections were included in each school’s folder along with transcripts and other documents.

#### Mealtime Observations

At each school site the research team conducted at least two observations of breakfast and lunch. Breakfast was typically served in the cafeteria and the classroom (if using an after the bell model); lunch was observed in the cafeteria. The observation goals were to capture rich data about the school food environment, practices and processes of serving meals to students, duration students had to eat, routines for entry and dismissal, and other important notes (see Additional file 3). These notes were included in each school’s folder and general mealtime observations/notes were integrated into qualitative interview procedures if appropriate to prompt discussion (e.g., “we noticed that staff played music in the cafeteria; whose idea was that?”).

## Data analysis

Aligned with the convergent mixed methods design of this study, we developed an innovative approach combining guidance from the CFIR authors [48] for deductive coding data scoring and recommendations from Guetterman et al. [49] for integrating qualitative and quantitative data in MAXQDA software using the TeamCloud interface [50].

## Step 1: Deductive coding

The structure of the interview guide and coding procedures outlined by CFIR authors [48] facilitated a deductive analysis approach, in that each question corresponded to a construct within each of the framework domains. We developed a coding system in MAXQDA that corresponded with the CFIR structure and uploaded all study transcripts, school demographic information, and other key variables into MAXQDA to allow qualitative coding. Following prior studies led by the PI using this process, [51] the research team met to develop a coding consensus document (Additional File 4), which described each CFIR construct and anticipated potential responses and themes that would emerge through the data. The CAB provided input on the consensus document and deductive coding procedures during the December 2023 and January 2024 meetings following demonstrations from the research team.

Coding transcripts comprised selecting and assigning key extracts from interview transcripts to a particular CFIR construct and adding comments showing rationale for coding allocation. Applying the CFIR systematic coding approach facilitated the assignment of numerical scoring to the qualitative data. Specifically, if a particular construct was deemed to have a positive influence on implementation given interview responses, a score of + 1 or + 2 was assigned for that construct. Conversely, if a construct was deemed to be a negative influence, a score of − 1 or − 2 was given. According to the CFIR rating system [48], the difference between ( ±) 1 and 2 depends on the strength of the data such that a score of 1 would indicate a moderate influence on implementation, whereas a score of 2 signals a stronger influence depending on the type of language used and the field notes taken by the research team from the live interview. For example, if a participant said they “really loved the menu and choices available for lunch”, this extract would be assigned a score of 2. Similarly, the research team sought clear examples in the data from participants to help make an informed decision. If a positive/negative influence not clear, a neutral score of 0 was given; a score of “X” was used for mixed results.

Scores were entered into a spreadsheet and into the comments on the MAXQDA coding system to enhance data-driven decision-making. The PI created a workflow document to guide qualitative analysis and scoring (see Additional File 5). To enhance credibility of analyses, the first five transcripts (one for each participant type from one school) were coded by each team member to ensure consistency in coding pattern, followed by ~ 20% of the transcripts being double coded by two of the five team members. Interrater reliability was calculated in MAXQDA through the coder agreement feature, and if < 75% agreement on construct coding occurred, the PI established consensus among the two coders to determine the final code. This iterative process continued for the first two rounds of coding, after which all disagreements were resolved through group discussions.

To prepare the quantified CFIR data for merging into the larger dataset, each participant ID was aligned with the scores for each construct and domain of the model. Any “X” scores (implying a mixed/uncertain rating) were converted to 0 for the purpose of analysis. Any constructs without a score remained blank so as not to misguide subsequent analyses. Quantitative CFIR scores, demographic data, and other pertinent data were imported into SAS Software [52] to generate descriptive statistics of the sample and subgroups. Following guidance from experts [53, 54] three coders used a consensus approach to assign an overall score to each school based on the mean score of each construct and the range of scores given across different participants within that setting. If mean scores were accompanied with a small score range, the mean score was rounded to the nearest whole number between −2 and + 2. In the case of large score ranges and where a mean score was generated from a small number of coded extracts, a score of 0 was given to signal a mixed/undetermined influence on implementation [53]. The PI led this process with the second and third authors since they were the most involved with qualitative coding.

## Step 2: Compiling scores and integrating quantitative data

As a team we did not want to be influenced by school meal participation rates in coding, so only after all qualitative codes were finalized and quantitative scores developed for CFIR constructs did we integrate participation data into MAXQDA. We obtained school-level breakfast and lunch participation data from the SDP and calculated mean participation rates from September-December 2023 [23], which spanned the course of data collection for the study. Participation is calculated for each month of the school year by dividing the number of meals served by the enrollment of all students in the school, and by the number of instructional days for breakfast and lunch respectively, yielding a percentage score for participation. After generating means for each school and given school characteristics, we reviewed the data and generated meaningful groupings of low, moderate, and high participation. These characteristics, along with other school-level variables (i.e., middle or high, full service or satellite) were entered into in MAXQDA to facilitate mixed methods analysis.

## Step 3: Examining determinants and areas of divergence between levels of participation

Using MAXQDA software we examined extracts for all CFIR constructs for the whole sample and compared Low, Moderate, and High participation groups. First, for Aim 1 to identify key determinants across the sample, the team selected the most salient positive and negative determinants from the sample to generate a quote matrix. This allowed the group to emphasize extracts from an array of participants discussing constructs and to interpret why these were scored more positively/negatively by reviewing the qualitative data.

For Aim 2, the team assigned an overall score for the 3 groups of Low, Moderate, and High participation based on the scores assigned in Step 2, and noted where divergence occurred among the three groups in terms of scoring that could help contextualize participation rates and identify specific challenges for low-participating schools. Examples included negatively scored constructs for low/moderate schools compared to positive mean scores for higher participation schools, or weaker positive scores in comparison. This facilitated the team’s focus on specific constructs that required further analysis. Following identification of key constructs, we created a joint display by generating a crosstabulation in MAXQDA to show coded extracts to the selected constructs, split by participation group. This allowed the team to review the coded segments according to participation group from a range of participant types; this facilitated more in-depth understanding of the constructs and allowed the team to identify key leverage points for Implementation Mapping.

## Qualitative rigor

Qualitative rigor must be upheld to ensure validity, reliability, generalizability, and confirmability of our qualitative analysis process [55, 56].

## Validity/Credibility

The team established a coding consensus document and logbook, which served as “living documents” that guided decision-making and alignment with qualitative coding. We took several steps to increase intercoder agreement among five different coders, which comprised each member coding the first five (of 121) transcripts to calibrate coding and scoring, followed by each team member coding 2 of another member’s assigned transcripts and the PI conducting agreement analyses in MAXQDA (% agreement), followed by the PI making executive decisions on coding discrepancies. Once the coders had > 75% agreement on the deductive coding, the team independently coded transcripts and conducted peer debriefing each week to modify documents and discuss coding interpretations. We conducted data source (i.e., field notes, observations) and participant (i.e., data from different participant types in each school) triangulation, which facilitated reflection and cross-referencing in coding. Observation data were utilized heavily to triangulate the interview data, especially where coders had areas of uncertainty or disagreement. Finally, our team spent time in the participating schools and were able to observe many practices and processes that took place, which helped interpretation of the data.

## Reliability/Dependability

The team kept an active audit trail in the MAXQDA TeamCloud logbook interface, which documented key decision-making. We also conducted regular peer debriefings in weekly team meetings from January-May 2024. Finally, to enhance our interpretation of the findings we regularly debriefed with CAB members who gave input on coding and analysis procedures, holding us accountable to confront our subjectivity and potential bias in coding.

## Generalizability/Transferability

In recruitment we considered the demographic characteristics (i.e., race and ethnicity, language spoken, household income and education) of our sample and compared them to those of the district. However, potential limitations in generalizability and transferability may arise due to sample biases and unique contextual factors in the schools who self-selected to participate in this research. Ensuring broader representation and considering context-specific influences are essential for drawing more comprehensive and applicable conclusions.

## Confirmability

Finally, to address confirmability, we took extensive field notes from interviews and school observations (and after virtual interviews if applicable). We utilized reflective practice in team meetings, using discussions to adapt coding definitions and inclusion criteria based on new data that challenged our positionality. Finally, CAB members’ feedback in developing local-level dissemination products helped us to synthesize data in a more transparent and meaningful way.

## Results

Eight schools across the SDP were included in this study (n = 6 middle schools, n = 2 high schools). Six of these schools had full-service kitchens, while two were satellite kitchens without the equipment to fully prepare and cook food at the school. Table 1 shows demographic information including participant role and race and ethnicity of the full sample and the characteristics by school. Aggregate data on food insecurity, participation rates, and attendance for each school is included from the SDP database.

**Table 1:** School characteristics for full sample and by participating schools

			**Participating Schools**															
	**Full Sample (N = 193)**		**1 (n = 19)**		**2 (n = 26)**		**3 (n = 12)**		**4 (n = 30)**		**5 (n = 28)**		**6 (n = 26)**		**7 (n = 35)**		**8 (n = 17)**	
**Variable**			**Grades K-8; SK**		**Grades K-8; FK**		**Grades K-8; SK**		**Grades K-8; FK**		**Grades 9–12; FK**		**Grades K-8; FK**		**Grades K-8; FK**		**Grades 9–12; FK**	
**Participant Type**	N	%	N	%	N	%	N	%	N	%	N	%	N	%	N	%	N	%
Middle School Student	27	14.0%	5	26.3%	1	3.8%	3	25.0%	3	10.0%	NA		6	23.1%	9	25.7%		
High School Student	20	10.4%	NA		NA		NA		NA		13	46.4%	NA		NA		7	41.2%
Parent	50	25.9%	7	36.8%	6	23.1%	2	16.7%	9	30.0%	2	7.1%	4	15.4%	19	54.3%	1	5.9%
Food Service	22	11.4%	1	5.3%	3	11.5%	1	8.3%	3	10.0%	5	17.9%	4	15.4%	1	2.9%	4	23.5%
Teacher	48	24.9%	3	15.8%	10	38.5%	3	25.0%	14	46.7%	3	10.7%	9	34.6%	3	8.6%	3	17.6%
Administrator	26	13.5%	3	15.8%	6	23.1%	3	25.0%	1	3.3%	5	17.9%	3	11.5%	3	8.6%	2	11.8%
**Race and Ethnicity**																		
American Indian/Alaska Native	3	1.6%	0	0.0%	1	3.8%	0	0.0%	0	0.0%	0	0.0%	0	0.0%	2	5.7%	0	0.0%
Asian	9	4.7%	2	10.5%	0	0.0%	0	0.0%	0	0.0%	3	10.7%	0	0.0%	1	2.9%	3	17.6%
Black/African American	80	41.5%	7	36.8%	14	53.8%	9	75.0%	10	33.3%	12	42.9%	13	50.0%	11	31.4%	4	23.5%
Hispanic/Latino	37	19.2%	7	36.8%	6	23.1%	0	0.0%	3	10.0%	7	25.0%	3	11.5%	9	25.7%	2	11.8%
Native Hawaiian/Other Pacific Islander	0	0.0%	0	0.0%	0	0.0%	0	0.0%	0	0.0%	0	0.0%	0	0.0%	0	0.0%	0	0.0%
Middle Eastern/North African	2	1.0%	0	0.0%	0	0.0%	1	8.3%	0	0.0%	1	3.6%	0	0.0%	0	0.0%	0	0.0%
White	48	24.9%	2	10.5%	3	11.5%	2	16.7%	17	56.7%	5	17.9%	4	15.4%	9	25.7%	6	35.3%
Self-identify	2	1.0%	0	0.0%	1	3.8%	0	0.0%	0	0.0%		0.0%	0	0.0%	0	0.0%	0	0.0%
NA/not provided	12	6.2%	1	5.3%	0	0.0%	0	0.0%	0	0.0%	0	0.0%	6	23.1%	3	8.6%	2	11.8%
**Secondary Data from the School District of Philadelphia**																		
** Meal Participation (Sept-Dec 2023)**																		
Breakfast Participation			55%		58%		65%		66%		39%		51%		43%		10%	
Lunch Participation			55%		63%		59%		47%		36%		57%		56%		33%	
Participation Group			Moderate		High		High		Moderate		Low		Moderate		Low		Low	
**Self-Reported Food Security**																		
High/Marginal			70.6%				71.2%		88.0%		56.4%				71.8%		63.0%	
Low			23.3%				23.1%		5.3%		20.0%				24.5%		28.4%	
Very Low			6.1%				5.8%		6.7%		23.6%				3.6%		8.6%	
**Attendance**																		
% with 90% + attendance in 2022–2023			64.52%		44.55%		50.93%		72.67%		49.79%		32.21%		59.86%		39.66%	

Note: 1) *FK* full-service kitchen, *SK* satellite kitchen. Food security data are gathered by the school district using the US Department of Agriculture screening tool in an annual survey. Data for school #2 and #6 are not available

Table 2 shows all participant demographic data. From the 8 schools, 193 participants participated in the study comprising teachers (28%), parents (25%), students (middle 14%, and high school 10%), administrators (13%), and food service personnel (11%). Participants identified as Black/African American (43%), white (26%), Hispanic/Latino (20%), Asian (5%), Middle Eastern/North African (2%), and other (4%). Most of the sample identified as female (69%) and reported English as their primary spoken language (84%). Of all adult participants, most reported their age between 30–50 years old (54%) and nearly all participants reported an education level of high school diploma or higher (98%). For caregivers, 72% reported being currently employed, and the average household income falls typically below $70,000 per year (87%). These demographic characteristics are close to those of the student body within the district [45] with a slightly higher percentage of participants identifying as white in our sample which may be due to our sample including parents, teachers/staff, and administrators.

**Table 2:** Participant characteristics (N = 193)

Variable	Frequency	%
**Participant Type**		
Middle School Student	27	14.0%
High School Student	20	10.4%
Parent	50	25.9%
Food Service	22	11.4%
Teacher	48	24.9%
Administrator	26	13.5%
**Race and Ethnicity**		
American Indian/Alaska Native	3	1.6%
Asian	9	4.7%
Black/African American	80	41.5%
Hispanic/Latino	37	19.2%
Native Hawaiian/Other Pacific Islander	0	0.0%
Middle Eastern/North African	2	1.0%
White	48	24.9%
Self-identify	2	1.0%
NA/not provided	12	6.2%
**Gender**		
Female (She/her/hers)	133	68.9%
Male (he/him/his)	53	27.5%
Neutral (they/them/theirs)	1	0.5%
Other	6	3.1%
**Primary Language**		
English	162	83.9%
Spanish	23	11.9%
Portuguese	0	0.0%
Chinese Mandarin	1	0.5%
Haitian Creole	1	0.5%
Vietnamese	1	0.5%
Arabic	1	0.5%
French	1	0.5%
Russian	0	0.0%
Bangla (Bengali)	0	0.0%
Other Language	3	1.6%
**Adult Participants (n = 136)**		
** Level of Education**		
Less than 8th grade	0	0.0%
Some high school	2	1.5%
High school diploma	34	25.0%
GED or alternate certificate	5	3.7%
Some college credit	11	8.1%
1 or more years of college	6	4.4%
Vocational/trade school	6	4.4%
Associates degree	8	5.9%
Bachelors degree	19	14.0%
Masters degree	43	31.6%
Doctoral-level degree	2	1.5%
**Age**		
17 or younger	7	5.1%
18–20	0	0.0%
21–29	19	14.0%
30–39	37	27.2%
40–49	37	27.2%
50–59	20	14.7%
60 or older	7	5.1%
N/A	9	6.6%
**Caregiver Participants (n = 47)**		
** Employment Status**		
Employed and working 1–39 h per week	20	43.5%
Employed and working 40 or more hours per week	13	28.3%
Not employed and looking for work	9	19.6%
Not employed and not looking for work	2	4.3%
Retired	0	0.0%
Disabled and not able to work	2	4.3%
Missing	1	2.2%
**Total Household Income**		
$0—$9,999	13	27.7%
$10,000—$19,999	7	14.9%
$20,000—$29,999	4	8.5%
$30,000—$39,999	5	10.6%
$40,000—$49,999	7	14.9%
$50,000—$59,999	2	4.3%
$60,000—$69,999	3	6.4%
$70,000—$79,999	0	0.0%
$80,000—$89,999	1	2.1%
$90,000—$99,999	0	0.0%
$100,000 or more	5	10.6%

## Aim 1 Findings

Table 3. shows the quantitative coding results for the overall sample and by participation groups. Given the nature of scoring and that in many cases the SD was larger than the mean, we present score distributions in the table and below to accompany the mean values. For the overall sample, the strongest assets/facilitators were Individuals – Mid-level leaders (M = 1.29 [−1,2]), High-Level leaders (M = 0.96 [−1,2]), and Implementation Process – Adapting (M = 0.97 [0,2]); the strongest negative determinants were Outer Setting – Market Pressure (M = −1.35 [−2,0]), Inner Setting – Relative Priority (M = −1.17 [−2,−1]), and Available Resources (i.e., Time (M = −1.10 [−2,2]). The right column shows the number of coded extracts across constructs to highlight which constructs were coded to the most versus the least. This informed the team’s approach in analyzing and interpreting data for Aim 2. Additional File 7 provides complete CFIR scoring for each school separately.

**Table 3:** Interview score descriptive statistics for domains and constructs for full sample and by participation level and extracted code frequencies

**Domain and Construct**	**Total Sample**			**Low Participation (3 schools)**			**Moderate Participation (3 schools)**			**High Participation (2 schools)**			Trend Observed ****	**Extracts Coded**
**I. Innovation Characteristics**	M*	Range**	Score***	**M**	**Range**	**Score**	**M**	**Range**	**Score**	**M**	**Range**	**Score**	(*)	**N**
A. Innovation Source	1.00	1	1	.	.	.	1	1	1	.	.	.		1
B. Innovation Evidence Base	0.33	(-2,2)	0	0.11	(-2,2)	0	0.78	(-1,2)	1	0	(-1,2)	0		22
C. Innovation Relative Advantage	.	.	.	.	.	.	.	.	.	.	.	.		0
D. Innovation Adaptability	-0.33	(-1,1)	0	.	.	.	0	0	0	-1	-1	-1		5
E. Innovation Trialability	-1.00	-1	-1	.	.	.	-1	-1	-1	.	.	.		2
F. Innovation Complexity	0.13	(-1,1)	0	-1	-1	-1	0.6	(-1,1)	0	-0.5	(-1,0)	0		5
G. Innovation Design	-0.65	(-2,2)	-1	-0.80	(-2,2)	-1	-0.49	(-2,2)	-1	-0.67	(-2,2)	-1		79
H. Innovation Cost	-0.86	(-1,0)	-1	-0.9	(-1,0)	0	.	.	.	.	.	.		2
**II. Outer Setting**														
A. Critical Incidents	-0.07	(-1,2)	0	0.17	(-1,2)	0	0	(-1,2)	0	-1	-1	-1		17
B. Local Attitudes	-0.59	(-2,1)	-1	-0.65	(-2,1)	-1	-0.39	(-2,1)	-1	-0.87	(-2,1)	-1		53
C. Local Conditions	-0.72	(-2,1)	-1	-0.56	(-2,1)	-1	-0.67	(-2,1)	-1	-1	(-2,0)	-1		47
D. Partnerships & Connections	-0.37	(-2,2)	0	-0.86	(-2,2)	-1	0.08	(-2,1)	-1	-0.25	(-2,2)	0		32
E. Policies & Laws	-0.93	(-2,0)	-1	-1	(-2,0)	-1	-1	-1	-1	-0.67	(-2,0)	-1		20
F. Financing	-0.33	(-1,0)	0	-0.5	(-1,0)	0	0	0	0	.	.	.		3
G. External Pressure	.	.	.	.	.	.	.	.	.	.	.	.		0
1. Societal Pressure	-0.71	(-2,0)	-1	-0.29	(-1,0)	0	-1.17	(-2,-1)	-1	-1	-1	-1		6
2. Market Pressure	-1.35	(-2,0)	-1	-1.38	(-2,0)	-2	-1.33	(-2,0)	2	-1.33	(-2,0)	-1		28
3. Performance Measurement Pressure	-0.18	(-1,1)	0	0	(-1,1)	0	-0.33	(-1,0)	0	-1	-1	-1		7
Procurement	-1.06	(-2,0)	-1	-1	-1	1	-1	(-2,0)	-1	-1.5	(-2,0)	-2		
**III. Inner Setting**														
A. Structural Characteristics	0.40	(-1,1)	0	-0.2	(-1,1)	-1	1	1	1	.	.	.		5
1. Physical Infrastructure	-0.47	(-2,2)	0	-0.71	(-2,1)	-1	-0.14	(-2,2)	0	-0.5	(-2,1)	0		36
2. Information Technology Infrastructure	0.83	(0,1)	1	1	1	1	0	0	0	.	.	.		1
3. Work Infrastructure	-0.49	(-2,2)	0	-0.59	(-1,2)	-1	-0.36	(-1,2)	-1	-0.5	(-2,1)	-1		29
B. Relational Communications	0.46	(-2,2)	1	-0.07	(-1,2)	0	1.19	(0,2)	1	0	(-2,1)	1	*	28
C. Communications	0.10	(-2,2)	0	0.36	(-1,2)	1	-0.125	(-2,2)	-1	0.09	(-1,2)	0		39
D. Culture	-0.16	(-2,2)	0	-0.19	(-2,2)	0	-0.33	(-2,2)	-1	0.32	(-2,2)	0	*	80
1. Human Equality-Centeredness	-0.47	(-2,2)	0	-1.43	(-2,-1)	-2	0.6	(-2,2)	0	-0.125	(-2,2)	1	*	28
2. Recipient-Centeredness	0.16	(-2,2)	0	0.28	(-2,2)	0	0.12	(-2,2)	0	0.06	(-2,2)	0		55
3. Deliverer-Centeredness	-0.66	(-2,2)	-1	0.33	(0,2)	0	-1.14	(-2,-1)	-2	-2	(-2,2)	-2		8
4. Learning-Centeredness	0.80	(0,2)	1	0.5	(0,1)	0	1.5	(1,2)	2	0	0	0		5
E. Tension for Change	-1.33	(-2,1)	-1	-1.83	(-2,-1)	-2	.	.	.	-0.33	(-1,1)	0		4
F. Compatibility	1.00	1	1	.	.	.	.	.	.	1	1	1		1
G. Relative Priority	-1.17	(-2,-1)	-1	-1	-1	-1	-1.17	(-2,1)	-2	-1.5	(-2,1)	-1		8
H. Incentive Systems	0.00	0	0	0	0	0	.	.	.	.	.	.		1
I. Mission Alignment	0.00	0	0	.	.	.	.	.	.	0	0	0		4
J. Available Resources	-0.90	(-2,2)	-1	-0.61	(-2,2)	-1	-1.12	(-2,0)	-1	-0.83	(-1,0)	0		45
1. Time	-1.10	(-2,2)	-1	-1	(-2,2)	-1	-1.12	(-2,1)	-1	-1.3	(-2,0)	-2		5
2. Funding	-0.50	(-1,1)	0	-1	(-1,1)	-1	0	(-1,1)	0	-1	-1	-1		13
3. Space	-0.37	(-2,2)	0	0.08	(-2,2)	-1	-1	-1	-1	-1.67	(-2,-1)	-2		42
4. Materials & Equipment	-0.14	(-2,2)	0	0.57	(-2,2)	0	-0.5	(-1,1)	1	-0.5	(-2,1)	-1		17
K. Access to Knowledge & Information	-0.24	(-2,2)	0	-0.4	(-2,1)	0	-0.29	(-2,2)	-1	0.14	(-1,1)	1	*	52
**IV. Individuals Domain**														
A. High-level Leaders	0.96	(-1,2)	1	1.38	(-2,2)	1	0.64	(-1,2)	1	1.33	(0,2)	1		23
B. Mid-level Leaders	1.29	(-1,2)	1	1.75	(1,2)	2	0.5	(-1,1)	1	1.5	(1,2)	2		14
C. Opinion Leaders	0.30	(-2,2)	0	0.36	(-1,2)	1	0.23	(-2,2)	0	0.4	(-1,2)	1		31
D. Implementation Facilitators	0.83	(-1,2)	1	1	1	1	0.89	(-1,2)	1	0	(-1,1)	0		11
E. Implementation Leads	0.79	(-1,2)	1	0.46	(-1,2)	1	1	(1,2)	1	1.33	(1,2)	2	*	16
F. Implementation Team Members	0.55	(-2,2)	0	0.67	(-1,2	1	0.45	(-2,2)	1	0	0	0		28
G. Other Implementation Support	-1.00	-1	-1	-1	-1	-1	.	.	.	.	.	.		3
H. Innovation Deliverers	0.48	(-1,2)	0	0.33	(-1,2)	0	0.27	(-1,2)	1	1.11	(-1,2)	2	*	25
I. Innovation Recipients	-0.38	(-2,2)	0	-0.47	(-2,1)	-1	-0.175	(-2,2	0	-0.61	(-2,-1)	-1		74
**Characteristics Subdomain**														
A. Need	0.89	(-1,2)	1	0.87	(-1,2)	1	0.79	(-1,2)	1	1.12	(-1,2)	1	*	62
B. Capability	-0.91	(-2,0)	-1	-1	(-2,0)	-1	-0.5	-1	-1	-1	-1	-1		6
C. Opportunity	-1.00	(-2,0)	-1	-1.11	-1	-1	-0.91	(-1,0)	-1	-1	-1	-1		19
D. Motivation	2.00	2	2	2	2	2	2	2	2	.	.	.		4
**V. Implementation Process Domain**														
A. Teaming	.	.	.	.	.	.	.	.	.	.	.	.		0
B. Assessing Needs	.	.	.	.	.	.	.	.	.	.	.	.		0
1. Innovation Deliverers	0.58	(0,1)	1	1	1	1	0	0	0	1	1	1		5
2. Innovation Recipients	-0.60	(-2,1)	-1	-1.08	(-2,1)	-1	-0.33	(-2,1)	0	-0.5	(-2,1)	-1	*	34
C. Doing	1.00	1	-1	.	.	1	1	1	1	.	.	.		6
D. Reflecting & Evaluating	.	.	.	.	.	.	.	.	.	.	.	.		1
1. Implementation	0.63	(-2,1)	1	0.57	(-2,1)	0	1	1	1	.	.	.		5
2. Innovation	.	.	.	.		.	.	.	.	.	.	.		0
I. Adapting	0.97	(0,2)	1	0.92	(1,2)	1	1	(0,2)	1	1	1	1		23

* Mean calculated from all scored extracts from school

** Range includes lowest and highest scores recorded for this sample/subsample

*** Score calculated by observing the mean, the range, and the number of responses for the construct to make informed coding decision

**** Indicates a change from - to + scoring between low, moderate, and/or high participation groups or meaningful difference in score

Table 4 shows results from MAXQDA quote matrices which comprise a selection of the most prevalent assets/positive determinants and needs/negative determinants found among the quantitative scoring of the interview data, alongside our coding protocol notes and associated interview extracts from an array of participants. Each quote/interview segment is accompanied by the participant type and whether they were at a low, moderate, or high participation school. Notably the primary assets/positive determinants relate to key implementing roles (i.e., administrators, implementation leaders) and inner setting, whereas most of the negative determinants predominantly reside in the outer setting.

**Table 4:** Primary Positive and Negative Implementation Constructs with Coding Notes and Interview Extracts

CFIR Construct	Valence ( ±)	Coding Consensus Notes	Extract
Mid-Level Leaders	+	Code: perceptions of whether/how school climate leaders/deans support school meals and how this impacts implementation	Some days, the dean will put music on that kind of keeps [students] a little mellow someone like dance to the music (Teacher, High Participation)
High-Level Leaders	+	Code: perceptions of whether/how school principals/administrators support school meals and how this impacts implementation	She been coming down to help clean the tables off a couple of days in. (Food service, High Participation)
Need	+	Code: statements highlighting food insecurity of students and families, and how the meals program alleviates hunger	Yeah, it's already a struggle out here, especially for these low income neighborhoods, the neighborhood schools, so the parents don't have to spend as much money. So that's. You know, everybody needs money around here. You know, everybody's either just making it or below, there's a lot of single mothers. So, with multiple children just doing it by themselves, so when we don't have to come out of our pocket to pay for food, it really takes the load off of the parents. (Parent, High Participation)
Adapting	+	Code: how (if at all) is the meals program being adapted and is this helping overall?	Because the way I do it with the menu that we have, I notice things they like for tomorrow we got yogurt and muffins. So normally instead of order on one case, I always get like two maybe three. So I know it's gonna be a little bit more (Food Service, Low Participation)
Market Pressure	-	Code: Any ongoing programs or initiatives that could pose a threat to school meal programs?	I feel as though that the corner store is more of a preference only because the child with the biggest bag is the most popular, you understand what I'm saying to you, oh, you have all of the snacks and you come with them every day. They more people gravitate towards them, you know, because they look at them, like, oh, they have money, or they have more things in the next person. So they're more popular, you know, so I feel like that is an influence. (Administrator, Moderate Participation)
Available Resources	-	Code: Statements that indicate there's not enough resources for implementation e.g. “The options for us to order are very limited” or from student perspective regarding choice Also “not enough food” is a limited resource, time, etc	Yeah, I don't think he gets enough time to eat and I don't think there's enough food because when he come home, he's still hungry. (Parent, Low Participation)
Innovation Design	-	Code: Issues related to clarity of information, quality of resources, food quality, preparation issues, portion size issues, perceptions of food quality	Maybe the presentation might be better. Yeah. Yeah, that could help. Yeah. Because if you look at a school lunch, you're instantly turned off. Yeah. It's disgusting looking. (Teacher, High Participation)
Local Attitudes	-	Code: perceptions of school meals across the whole school community and alignment with culture/background of families/community members	During black history they gave out chicken, what kind of chicken this is, was it? It was like it was like, No, it was baked. It was baked. Like as soon black history they just gave out chicken. (Student, Low Participation) Do you guys feel that your backgrounds are reflected in the food at all? No I don't feel like it's reflected at all. (Student, Low Participation)
Local Conditions	-	Code: factors within the local area/neighborhood affecting implementation of food service, socio-economic conditions of families around the school, etc. that might impact implementation	Well, there's not really a lot of healthy places around here there's a pho the pho place and then there's a sushi, sushi. But most of the most of the food we have here come from like a Rite Aid or Walgreens or the popeyes. (Student, Low Participation) For example, people have mentioned, like, kids coming to school with stuff from the [corner] store, rather than sitting there eating that rather than school meals (Administrator, Low Participation)
Policies and Laws	-	Code: factors related to district, state, or federal policies affecting school meal implementation	The paperwork is tremendous. But other than that, I'd rather have more time to spend with the kids and talking to them about what they would like to see on the menu (Food Service, Low Participation)
Relative Priority	-	Code: The degree to which school meals are viewed as a priority in school operations, or “take a back seat” in terms of programming due to other pressures such as other after school programs/classroom instruction	Like I said, we don't have a gym. We just lost two teachers. You know, we have a condemned trailer, I'm trying to get removed from the yard, like, so every teacher has a list every principal has a list like this. And so then when they're thinking about the institutional issues of food, like they're, it's just down the list, right. (Administrator, Moderate Participation)

Valence = directionality of the determinant i.e., positive (support/facilitator) or negative (challenge/barrier)

For all schools, it was clear that the administrators/deans of students, and Mid-level leaders such as school climate leaders (responsible for coordinating recess/meal operations in communal spaces) were the biggest facilitators and for the most part got involved to support operations. Administrators can impact school meals by supporting food service with operations, hiring climate staff to help facilitate implementation and build social culture in the cafeteria, and modifying schedules to allow more time for meal consumption. Climate leaders (mid-level) are responsible for overseeing recess and mealtimes through behavior management in the cafeterias and playgrounds, helping to promote school meal participation, among other key roles. Further, despite challenges faced, the level of adaption made among front-line implementers to meal service operations (e.g., modifying schedules, ordering food items that are popular to ensure there’s enough food) were noted as strengths across the school settings. Finally, the level of need/dependency on school meals to mitigate food insecurity was overwhelmingly coded as a positive determinant, showing the overarching support for this program in school settings but highlighting the deprivation among families driving such need.

Some of the most negative determinants from the Outer Setting were Market Pressure (i.e. how much do school meals compete with outside foods being brought in?), Local Attitudes (i.e., shared beliefs of students and families around meals/alignment with community culture), Policies and Laws (i.e., district, state, or federal regulations that impact food service), and Local Conditions (i.e., safety of surrounding area, access to healthy foods outside school). Specifically, participants talked about the lack of alignment of the school menus with student/family culture which may pose challenges for participation. This, coupled with overarching challenges to accessing healthy food/heavy prevalence of corner convenience stores, limits students’ exposure and socialization to balanced school meals.

Further, related to innovation design, many concerns were raised about the quality and appearance of school meals from a wide array of participants, which could play a significant role in their choice to participate. Policies and laws affecting implementation (and therefore reach) include the ways schools (and the district) must comply with USDA regulations on portion size, calorie count, and ingredients used for all meals served, limiting their capacity to adapt the menus within the available budget to appeal to student preferences. Moreover, for a meal to be reimbursable, students must select each item offered, which may deter some students from participating if they do not like the food offered that day. Finally, the lack of resources such as time and space were prevalent across the whole sample, with students lamenting a lack of time to eat a full meal and being rushed, in addition to scheduling breakfast and lunch too early/late in the school day.

## Aim 2 Findings

We noted several constructs that seemed markedly different among low, moderate, and high participation groups whereby the quantitative score notably increased from low–high groups: Culture (i.e., social culture around school meals); Access to Knowledge and Information (i.e., school menus, training and support); Implementation Leads (i.e., food service managers); Human Equality Centeredness (i.e., ensuring equitable access to food and decision making); and Assessing Needs—Innovation Recipients (i.e., involving students and parents in decision making). Except for assessing needs (implementation process domain), these constructs all reside within the inner setting, which indicates differences in participation may be impacted by school-level decisions and policies. Table 5 shows the joint display created through crosstabulation tools in MAXQDA, which highlight extracts for each construct across the three different groups from a range of participants.

**Table 5:** Joint Display of Diverging Implementation Constructs and Key Qualitative Extracts by Participation Level

		**Qualitative Extracts**		
**CFIR Construct**	**Summary of Differences among Participation Levels**	**Low Participation (< 50%)**	**Moderate Participation (50–60%)**	**High Participation (> 60%)**
Culture	Overall informants from the lower participation school reported experiencing more stigma/discrimination among students who participated in school meals	I'm never thinking about it. I feel like there's a lot of kids getting food, but I guess there's all those kids that aren't. I don't know some kids are funny they want to put the hoods up and stuff because they don't want to get seen getting a free meal…They'll be like "hurry up hurry up. I don't want to be [seen]." Not that many. I guess whoever’s seeing them getting a free meal. (Administrator) They just feel embarrassed about eating a eating a freebie. That's what we call em. (Food Service) Sometimes people joke around and they be like, "Oh, you eating school lunch so like you homeless or whatever" (Student)	But kids know that this is like a safe place. And you can tell like, overall, when I'm in another classroom, it's just the relationship between the kids and the teachers and the report everybody like in the halls and so it's improved. It's a it's a I think, a very positive climate, even in the lunch room and in an in recess, which is like the hardest places (Administrator) I would take a free meal. Nobody got to say nothing to me. (Student) Because it is like kind of 5050 I don't think anybody's like worried or ashamed to go get the school lunch versus pack a lunch or anything like that. It seems pretty. Pretty much like a safe environment. When I've been in the cafeteria, it seems like a comfortable environment for everyone. (Teacher)	I like to eat in the cafeteria because then I get to be with my friends and stuff. Yeah. And then we can have like a little conversation. And then when it's time is up, then we just stop our conversation and go to class (MS Student) Some of my friends like to eat free food. They're like, Oh, you're eating and freebies. But I think they like it. So I'm not going to judge a book by its cover, like people like different things. Yeah. So I'm not gonna say nothing mean (Student) I don't personally see that in my classroom. But that's also because I don't like no one in my classroom has been shamed for taking the breakfast. You know, I've also created the expectation that if you are hungry, like grab a snack, you can grab a snack, you're gonna you're you are you are 12, you are growing. (Teacher)
Access to Knowledge and Information	Issues regarding communication and information sharing appeared more prevalent in low-participation schools whereas in higher participation schools participants said the menu was easy to find	Interviewer: Is there anything regarding the climate PD that kind of focus on school meals? "No, I've never been in the PD, that are focused on it."—Climate Staff "Like I said, they used to send the menu, which they don't anymore. Now, they don't send them anymore. They didn't use this in them every month, but they stopped." (Parent) So they send it so they send it home. Again, I don't think they sent them home this month. (Teacher)	Okay, we're not set the lunch menu, we're only sent the breakfast menu. So I don't know what's coming up for lunch on any given day. And I don't really think the students are aware of it either. (Administrator) I don't even know where to access the lunch menu. Okay, we're not set the lunch menu, we're only sent the breakfast menu. So I don't know what's coming up for lunch on any given day. And I don't really think the students are aware of it either. (Teacher)	Not because everything is actually on the computer, anything parents want to know. You know, to count the calories was in the food, everything. They can look it all up on the Food Service website. (Teacher) Everything is actually on the computer, anything parents want to know. You know, to count the calories was in the food, everything. They can look it all up on the Food Service website. (Food Service)
Implementation Leads	Food service managers seemed to be more active and engaged in implementation among moderate/higher participation schools	Okay, this is a brand new cafeteria manager. So I can't speak to this year, think most of it's, he's just learning how to order properly and too much and how many and things like that. (Administrator) Because I remember not getting into an argument with the food service manager. But inquiring why there is no milk for breakfast when kids want milk. And she was I will send them down if they want milk. No, that means you're gonna have kids out of their classrooms and in the hallways and you fear all these questions that we have, we're doing that for each classroom. That's like 20 Plus kids out of the hallway is just too much. So I don't know if that's a logistical thing. (Administrator)	And you know, the fact that it's prepared daily, [food service manager] gives them choices in between the salads and the sandwiches and you know, having a variety on the menu (Teacher) I mean, I think it's I think it's between 60 and 70% [participation]. Nice, which is nice. And you know, the fact that it's prepared daily, she gives them choices in between the salads and the sandwiches and you know, having a variety on the menu (Administrator)	And sometimes what I'll do is, I'm entitled to one meal a day. We all are food service. So I'm a vegetarian. So if I'm not eating fruit, I'm not eating any of this. Yeah, and I'll offer my meal to another kid (Food service manager) Some time, I might have gifts for them. For the class, they who know their ID numbers, I get them little gifts, stuff like that. Just keep them wanting to come to lunch and eat the lunch, you know. (Food Service Manager)
Human Equality-Centeredness	Challenges in making sure all students have the same menu options seemed more prevalent in lower participation schools	There's a whole line of kids and if you're one of the last people in line it's hard for you to get what [meal] you want. (Student) Sometimes people get what you want. Sometimes you get what you don't want like sometimes it's just like this is like sometimes a cycle because you have a whole line of kids and like You're like one of the last people in line so it's like hard for you to get what you want. (Student) It's not equitable distribution for food either. And I would say even from elementary to middle, if you're noticing an order High School, like either it's just not an equitable situation when it comes to food service (Teacher)	And I don't think you're talking about equity. Like across the district it's not the same, like what we have is not what sure it's not what's in [wealthier neighborhood]—they probably have like a nice full kitchen. (Administrator) Sometimes when they get down there, I guess because they're in the later lunches. The most preferred food is already gone. So now they're stuck with the food that they don't like. So then situation after school where I'll have to, you know, go to the store and buy them a sandwich or something like that, so that they're not hungry (Parent)	For breakfast each month they switch the grades that are in the lunch room, so they have some eat breakfast in the lunch room and then some eat breakfast in the classroom. And they switch it up every month. So that's kind of all right. (Parent) You know, if I'm getting a space meal and you're getting a space meal, we both got the same space meal. Yeah, it's not, I gotta have cheese and fries, and you got to treat to steak and fries. And you got a hamburger and fries. It's we all got the same (Administrator)
Assessing Needs—Innovation Recipients	Students and parents felt they had more of a voice in meal implementation in moderate/higher participation schools	(But do your children have any say at all in what served for lunch? Does the school ever ask them?) "I don't think so." (Parent) No, no. like they don't ask you (Student) But you feel like you don't have a say because I feel like I can't say nothing since it's free. (Student)	No, because they don't even ask us what we want. They just give it to us.(Student) Yeah, that's the only question. And that's a misleading question. You know, because I mean, you're providing food for people. I think it's not the food security is the issue. But that's not the that's not the main issue. You know, it's like, are your food needs being met is the question? (Administrator)	I feel like as far as, like the students, they're meeting with the principal like we have a student council advisory principal advisory. And there's one student in my class, she did take the poll, she did her notes. And she asked the students what they like or dislike, and everybody says the same thing, but lunches, that was one of the things so hopefully, when it goes to the principal, they'll see about what can be done (Teacher) Even what they did when they created [online platform to show the menu]. Last year, we did have a QR code on a flyer. So that allowed the students to go on there to make suggestions, because they constantly complained to food service servers. And I said to them, you have a digital voice, let it be heard. Go on the website. Tell them what you like and what you don't like (Food Service)

In relation to culture, stigma and discrimination among students and observed by parents/adults in the school setting was a global issue among all schools. However, the high participation schools did not report quite as much stigma in their settings as the others evidenced by extracts coded less strongly as in moderate and low participation groups (i.e., 0 or −1 compared to −2). Access to knowledge and information presented as a challenge specifically in low-participation schools whereby participants reported not being able to access the school meals menu or being able to find out what was being provided at school, which was a frustration among parents and teachers. In the moderate and higher participation schools, the active role that food service managers played in day-to-day activities and their passion for their roles was noted, which may relate to how well schools are able to implement. Further, related to human equality-centeredness, the low participation group was coded much lower than the others and some examples manifested where participants felt not everyone had equal opportunity to access the same meals. For example, in one school the lunch schedule meant that the same grade levels were last to receive meals and menu options were not always still available to grades with later lunch times. Finally, and related to human equality-centeredness, the assessing needs – innovation recipients construct was coded negatively across all groups but most in the low participation group. Overall, students felt their voices were not heard regarding the menu or other aspects of food service, potentially leading to disenfranchisement. This was less of an issue in the higher participation schools but something that was evident in each setting.

## Discussion

The purpose of this study was to elucidate the primary determinants of USM implementation and participation, and to assess key challenges and supports across schools with varying levels of USM participation. This study comprises the first step of an ongoing Implementation Mapping process in collaboration with the SDP and a diverse CAB, and to our knowledge is among the first studies to utilize a mixed methods approach grounded in health equity and implementation science frameworks that is truly embedded within the community. Findings highlighted key supports to implementation which centered mostly on school leaders and food service providers, yet challenges related to equity and policy constraints were prevalent in the data. We observed differences among low, moderate, and high participation schools such as the degree to which students felt involved in decision making, prevalence of stigma and discrimination in participating in USM, and human equality-centeredness. The findings specific to stigma contradict recent research conducted with students in California [57], and prior literature [57–59], which warrant further consideration in future research.

The involvement of Mid-level and High-level leaders emerged as a significant positive determinant of program success. Additionally, the high level of dependency on school meals to address food insecurity underscores the critical importance of these programs in supporting vulnerable students and families, reflecting prior work addressing the impacts of USM [16, 60]. Administrators, deans of students, and school climate leaders played crucial roles in facilitating operations, demonstrating adaptability in managing meal service logistics. This was not a surprising finding, and reflects a strong body of literature on the importance of food service managers [46, 61] but adds insights about the role of climate staff, deans of students, who may be an underutilized asset in USM implementation. Although a lack of research on the role of mid-level managers in school settings exists, we see our findings reflected in research conducted of these representatives in implementation within healthcare [62]. The authors highlight that mid-level managers can shape the implementation climate, but more research is needed to understand determinants of involvement from these representatives.

Several significant barriers were identified, primarily within the outer setting. Market pressures, local attitudes, and policies/laws were major negative determinants, affecting the alignment of school menus with the cultural preferences of students and families. Participants highlighted challenges of corner convenience stores that are highly prevalent in urban low-income settings [63, 64], which limit students' exposure to balanced school meals, and the poor quality and appearance of school meals, which could deter participation. These barriers specifically point to issues of equity in implementation [34] that have seldom been highlighted in prior USM research. Thus, to provide equity-focused implementation strategies to improve USM uptake, these primary barriers must inform the co-development process with intervention schools and their districts. For example, menus could be revised to better integrate the cultural backgrounds of families, educational materials and learning sessions could be held to discuss the importance of nutritious school meals over purchasing foods from convenience stores, and/or more decision-making power could be given to students and families regarding USM implementation. Resource limitations, including insufficient time and space for meals and inconvenient scheduling, were prevalent across the sample. This finding has been cited as the main barrier for optimal USM implementation [46, 60, 65], and should be considered in USM implementation. Finally, outer setting factors such as market pressures are more difficult to address given a lack of control from school settings. Interventions have been conducted to improve access to healthier foods in corner stores [66, 67], yet to date no documented efforts to engage corner store owners and schools to develop solutions for meal participation are available, warranting further consideration. We plan to engage with these representatives in addition to non-profit organizations working with them (i.e., the Food Trust) in the next phase of our implementation mapping process.

The study revealed notable differences among low, moderate, and high participation groups in several constructs. High participation schools reported less stigma related to school meals, suggesting that a positive school culture can enhance participation rates. Since the adoption of the community eligibility provision, researchers have hypothesized a reduction in stigma [58, 68], but of the limited qualitative research to date, USM may not have the intended impact on reduction [69, 70]. This highlights a critical issue that may inform the development of a USM implementation strategy or compilation of strategies and relates specifically to the equity-related issues discussed above for the whole sample. For example, the lower participation schools may need more intense strategies that focus on changing the culture of school meals, involving the broader school community and centering student input and voice. For the higher participation schools, less rigorous support may be sufficient and instead providing evaluation and audit and feedback strategies to amplify what’s working in their systems.

Access to knowledge and information was a significant challenge in low participation schools, where participants struggled to obtain information about school meals. Conversely, in moderate and high participation schools, food service managers played an active and passionate role in day-to-day activities, potentially contributing to better implementation outcomes. Prior research highlights the important and underappreciated roles of food service managers [65]; their leadership in developing and executing an implementation strategy could have a significant impact in the next stages of Implementation Mapping. Issues of human equality-centeredness were more pronounced in low participation schools, with reports of unequal access to meals and scheduling disparities. Involving students and parents in decision-making was more common in high-participation schools, emphasizing the importance of community engagement in fostering program success. Overall, youth engagement in research on programs which ultimately affect them is lacking [71, 72], and the students’ perspectives about stigma and wanting more input in school meals provided critical information that can drive development of USM implementation strategies.

## Limitations

This study offers valuable insights into the implementation of USM. However, several limitations should be acknowledged. First, although the study included a diverse participant pool from eight schools in a large, urban school district, the findings may not be fully representative of all schools within the district or other districts with different demographics and contexts. Second, the identified implementation determinants are specific to the SDP and may not be directly applicable to other regions with different policies, cultural contexts, and resources. The unique challenges related to market pressures, local attitudes, and resource limitations might vary significantly in other settings. However, it must be noted that globally school meal programs are increasing, specifically in low and middle-income countries, and local governments have increased funding to support USM-like policies [19]. Thus, the Implementation Mapping process and methods in this study can be applied to emerging work domestically and globally. Finally, the study involved 193 participants, but the proportion of students (both middle and high school) was relatively low compared to teachers, parents, administrators, and food service personnel. This imbalance could skew the findings towards adult perspectives and may not fully capture the experiences and needs of the student population, who are the primary beneficiaries of the USM program.

## Conclusion

This study provides valuable insights into the implementation of school meal programs in the SDP. The purposeful collaboration with a CAB enhanced a more reflective and intentional analysis process, which made us change and adapt coding procedures based on feedback. Although the involvement of dedicated leaders and the adaptability of front-line implementers were significant facilitators, various barriers related to market pressures, cultural alignment, and resource limitations hindered program effectiveness. Addressing these barriers through targeted strategies, such as enhancing communication, fostering a positive school culture, and ensuring equitable access to meals, is essential for improving participation and outcomes. The next steps of this Implementation Mapping research should continue to explore these dynamics and develop tailored interventions to support the success of school meal programs in underrepresented settings.

## Supplementary Information

Additional File 1: Recruitment Flyer in English and Spanish

Additional File 2: Qualitative Interview Guides

Additional File 3: Observation Sheet

Additional File 4: Coding Consensus Document

Additional File 5: Qualitative Coding Protocol

Additional File 6: Complete CFIR Scoring Table

Additional File 7: COREQ Checklist

## Data Availability

Data associated with this manuscript can be requested from the corresponding author.
