# Supplementary material for: Understanding implementation determinants of universal school meals through an equity-driven mixed methods approach
Source: Implement Sci Commun. 2025 Apr 15;6:44. doi: 10.1186/s43058-025-00713-0 (PMC12001678; doi:10.1186/s43058-025-00713-0)
Supplement: Supplementary file 1 — Additional File 1: Recruitment Flyer in English and Spanish [file 43058_2025_713_MOESM1_ESM.pdf]

Philadelphia School Meals Collaborative

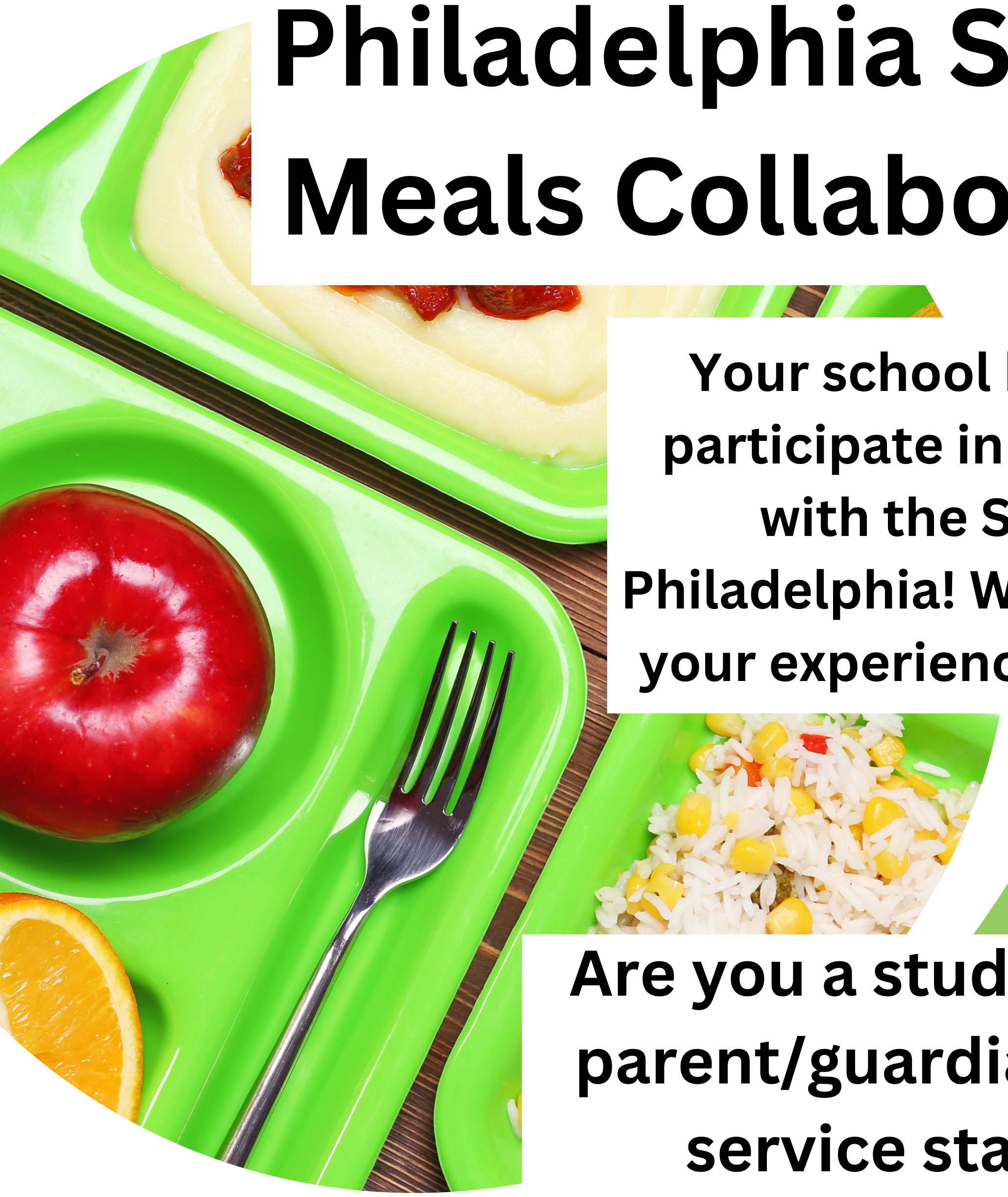

Your school has been chosen to participate in a needs assessment with the School District of Philadelphia! We want to know about your experiences with school meals

Are you a student (grades 6-12), parent/guardian, teacher, food service staff/manager)? Would you like to earn a $15 gift card for giving us feedback?

National Institutes of Health

Scan here to sign up to participate: **https://redcap.link/needsassessmentsurvey**

Temple University College of Public Health

Questions? Email our team at implementationlab@temple.edu

Escuela de Philadelphia Comidas colaborativas

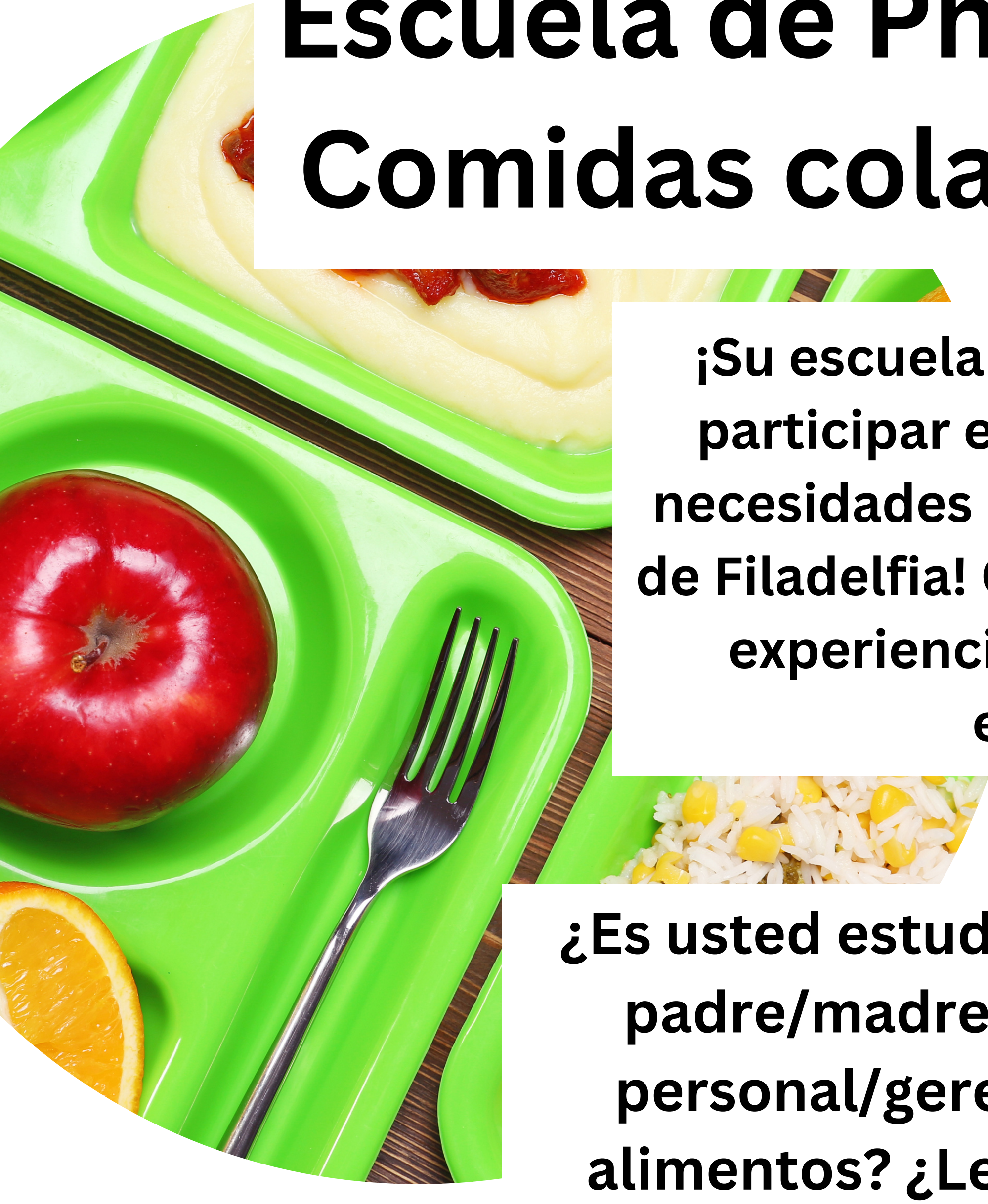

¡Su escuela ha sido elegida para participar en una evaluación de necesidades con el Distrito Escolar de Filadelfia! Queremos conocer sus experiencias con las comidas escolares.

¿Es usted estudiante (grados 6 a 12), padre/madre/tutor, maestro/a, personal/gerente de servicio de alimentos? ¿Le gustaría ganar una tarjeta de regalo de $15 por compartir su opinión?

Escanee aquí para registrarse y participar:
https://redcap.link/needsassessmentsurvey

NIH
National Institutes of Health

Temple University
College of Public Health

¿Preguntas? Envíe un correo electrónico a nuestro equipo a implementationlab@temple.edu
