# Supplementary material for: Understanding implementation determinants of universal school meals through an equity-driven mixed methods approach
Source: Implement Sci Commun. 2025 Apr 15;6:44. doi: 10.1186/s43058-025-00713-0 (PMC12001678; doi:10.1186/s43058-025-00713-0)
Supplement: Supplementary file 2 — Additional File 2: Qualitative Interview Guides [file 43058_2025_713_MOESM2_ESM.docx]

**Student Focus Group Guide**

**Introduction:**

Hi, my name is [insert name] and I'm from [insert organization]. Thank you for agreeing to talk with me today. I'd like to ask you a few questions about your thoughts on school meals. The information you share will be used for research purposes and to inform people working in your school and across the city of Philadelphia about how best to offer school meals. We'll combine all the results, and you and your school will not be identified with your responses. Your participation is voluntary, and if you don’t feel comfortable answering a specific question, you don’t have to. This should take about 30-45 minutes; you can stop anytime. There are no right or wrong answers. We just want to know what you think. If you have any questions, you can talk with the study's Principal Investigator, Gabriella McLoughlin. The contact information is in the consent form. I'm also planning to record our conversation. We'll delete the recording after we've written down your answers. And again, you won't be identified with your responses. Is it ok with you if we record this interview?

**Background and icebreaker:**

1. First, can we go around the group and say your name and remind me what grade you are in?
2. (Icebreaker) Let's start with an easy question. Can you tell me about your favorite foods and why those are your favorites?

[Construct: Innovation domain - Universal school meals (USM) characteristics]:

1. How long have you been at this school?
2. What are your favorite things about school meals? What about your least favorite?
3. How much of a say do you get in breakfast and lunch at your school?
4. How would you describe the healthiness of the meals currently served at school?
5. How much do you value school meals being healthy? Could you explain why?
6. How similar or different are the foods served at school compared to the foods you eat at home or where you go after school?
   1. What foods do you typically eat at home? On weekends?
   2. [If they mention differences, probe about the differences]: How is it different? Are the differences mainly in the types of foods, how they're prepared, or something else?
   3. What types of foods that you eat at home would you like to see served at school?
   4. How many students at this school eat school meals? (take a guess)
7. What would you change about the school meals?
8. Timing
9. Food options
10. Other?

[Construct: Outer Setting – Neighborhood characteristics]:

1. When you think of the school and surrounding area, what barriers are there to eating healthy?
2. (if they mentioned that food is different at home versus school – probe) so it seems there are differences between your family food environment and what the school provides. Can you maybe tell me why you think this happens?
   1. Probe on culture, diet, etc
   2. Neighborhood factors (i.e., food swamp or food desert)

[Construct: Individuals Domain – Innovation Recipients]

1. Philly is a really diverse city with lots of different cultures and neighborhoods. If you can think about your neighborhood and background, how do you see this reflected at school?
   1. How do you see your background supported at school (if at all)? What about at breakfast and lunch?
2. How could your school meals be better suited to the students at this school?
3. What can this school do to better serve its students? How can we make students feel more welcome and supported in eating school meals?

[Construct: Inner Setting – social culture around meals and stigma etc.]

1. So every school in Philly provides free breakfast and lunch to students. But we would like to learn how do students who participate in free school meals get treated at this school? How do you feel as someone who eats school meals?
2. How much interaction do you have with food service staff? How are these interactions?
3. What are your perceptions of the cafeteria? How do you feel welcome (or not) in this space?
4. Could you describe what the cafeteria environment is like when you walk in?
5. Can you share how you feel when it's time to eat school lunch? When I say feelings, I'm asking whether you feel, for example, happy, sad, excited, grateful, embarrassed, self-conscious, or any other emotion. [PROBE: Can you tell me more about it?]
6. How do the opinions of others influence your choice to eat or not eat school meals? [PROBE: Have you ever felt embarrassed or pressured? why did you feel that way?]
   1. Do you think other students at your school might feel embarrassed or are less comfortable eating school lunch? Why do you think they [feel/ don’t’ feel] embarrassed?
   2. [PROBE: Why do you think they might feel [uncomfortable / comfortable] eating school breakfast/lunch? What reasons could contribute to their feelings?]
   3. How do you think your classmates perceive eating school meals? [PROBE if needed: What kind of attitudes or feelings have you noticed from your friends or classmates about eating school meals?
   4. Have you noticed any stigma, peer pressure or judgment related to eating school meals at your school? [PROBE: Could you share some examples or explain a bit more?]
   5. What reasons could contribute to the [stigma/ lack of stigma] related to eating school meals?

**Wrap up:**

- That was the last question I have.
- What didn’t I ask about that you would like to share about school meals?
- Or anything you would like to ask me?
- Thank you so much. To thank you for your time, we will send you a gift card for $25 if you indicated in the survey that you would like to receive one.

**Caregiver Focus Group Guide**

**Introduction:**

Hi, my name is [insert name] and I'm from [insert organization]. Thank you for agreeing to talk with me today. I'd like to ask you a few questions about your thoughts on school meals. The information you share will be used for research purposes and to inform people working in your school and across the city of Philadelphia about how best to offer school meals. We'll combine all the results, and you and your child’s school will not be identified with your responses. Your participation is voluntary. This should take about 30-45 minutes; you can stop anytime. There are no right or wrong answers. We just want to know what you think. If you have any questions, you can talk with the study's Principal Investigator, Gabriella McLoughlin. The contact information is in the consent form. I'm also planning to record our conversation. We'll delete the recording after we've written down your answers. And again, you won't be identified with your responses. Is it ok with you if we record this interview?

**Background/context:**

1. (if a focus group) Can you please say your name and your child’s name, and what grade they’re in?
2. How long has your child been at this school?

[Construct: Innovation Characteristics]:

1. What are your child’s favorite things about school meals? What about their least favorite?
2. How much of a say do you and your children get in breakfast and lunch at your school?
3. What would you/your children change about the school meals?
   1. Timing
   2. Food options
   3. Other?
4. How much interaction do you have with food service staff? How are these interactions?
5. What are your child’s perceptions of the cafeteria? How do they feel welcome (or not) in this space?

[Construct: Individuals Domain – Innovation Recipients]:

1. What’s your approach to mealtimes at home? What does a mealtime look like in your household?
2. Philly is a really diverse city with lots of different cultures and neighborhoods. If you can think about your neighborhood and background, how do you see this reflected at your child’s school?
3. How could school meals be better suited to your child and other students at this school?
4. To your knowledge, how do students who participate in free school meals get treated at this school?
5. What can this school do to better serve its students? How can its staff make students feel more welcome and supported in eating school meals?

[Construct: Inner Setting – culture around school meals, stigma etc.]

1. How do the opinions of others influence your child’s choice to eat or not eat school meals? [PROBE: Have they ever felt embarrassed or pressured?]
2. Do you think other students at your child’s school might feel embarrassed or are less comfortable eating school lunch? Why do you think they [feel/ don’t’ feel] embarrassed?

[PROBE: Why do you think they might feel [uncomfortable / comfortable] eating school breakfast/lunch? What reasons could contribute to their feelings?]

1. How do you think your child’s classmates perceive eating school meals? [PROBE if needed: What kind of attitudes or feelings have you noticed from your friends or classmates about eating school meals?
2. Have you noticed any stigma, peer pressure or judgment related to eating school meals at your child’s school? [PROBE: Could you share some examples or explain a bit more?]
3. What reasons could contribute to the [stigma/ lack of stigma] related to eating school meals at your child’s school?

**Wrap up:**

- That was the last question I have.
- What didn’t I ask about that you would like to share about school meals?
- Or anything you would like to ask me?
- Thank you so much. To thank you for your time, we will send you a gift card for $25 if you indicated in the survey that you would like to receive one.

**Principal/Teacher Interview Guide**

**Introduction:**

Hi, my name is [insert name] and I'm from [insert organization]. Thank you for agreeing to talk with me today. I'd like to ask you a few questions about your thoughts on school meals. The information you share will be used for research purposes and to inform people working in your school and across the city of Philadelphia about how best to offer school meals. We'll combine all the results, and you and your school will not be identified with your responses. Your participation is voluntary. This should take about 30-45 minutes; you can stop anytime. There are no right or wrong answers. We just want to know what you think. If you have any questions, you can talk with the study's Principal Investigator, Gabriella McLoughlin. The contact information is in the consent form. I'm also planning to record our conversation. We'll delete the recording after we've written down your answers. And again, you won't be identified with your responses. Is it ok with you if we record this interview?

**Background/Context:**

1. First would you please say your name and position?
2. How long have you been this school’s principal/teacher (in X grade)?
   1. How have you found this role so far? (feel free to probe on things they like/dislike about their job)
3. Can you tell me a little about your school’s climate? How is attendance at your school?

[Construct: Innovation Domain - Universal school meals (USM) characteristics]:

1. Can you tell us about breakfast and lunch service at this school?
2. What is your observation about students’ breakfast habits? What do you think students eat before they come to school, at home or on the way? What do you think about that?
3. What are your observations about students’ lunch habits? Do you see patterns or trends in participation in lunch? What are they and do you have any perceptions of influential factors to participation?
4. Do you yourself eat school breakfast or lunch? If so how regularly?
5. What do you think about breakfast and lunch service at this school?
   1. Follow-up on food quality separately.
   2. Follow-up on anything they might say about school climate, student achievement, views about the neighborhood/community/parental engagement, partnerships.

[Construct: Individuals Domain – students as innovation recipients]

1. Can you tell me a little about your students and their lives outside of school? To your knowledge, do families at this school struggle with food insecurity? Other health challenges?
2. Do you observe students experiencing hunger, especially in the morning? What do you do?
3. What common comments do you hear about breakfast and lunch service from students?
4. How do you think parents view school breakfast and lunch service?
   1. To what degree are community members aware of/engaged in conversations about school meals?
5. What factors do you think are important to consider in promoting school meals to students/families?
   1. Demographics (i.e., race/ethnicity)
   2. Cultural factors (i.e., religion)
   3. Other?
6. To what degree do you think school meal programming is designed with these factors in mind currently?

[Construct: Inner Setting – social culture around meals and stigma etc.]

1. How would you say the culture of school meals is within your school?
2. Who makes decisions about breakfast and lunch service at your school and how? (Both ordering the food itself and delivery model)
3. What common comments do you hear about breakfast and lunch service from your teachers and staff?
   1. Follow-up on custodial staff and lunchroom staff.
4. How do decisions about school meals typically get communicated between lunch service staff and teachers/administrators?
5. What training (if any) is provided to promote awareness/engagement around school meals?
6. To what degree do you think the school culture influences/is influenced by school food service?

[Construct: Outer Setting]

1. Can you tell us about some of the city/district policies that may influence how school meals are served?
   1. What sort of an impact do they have?
2. What kinds of budgetary/financial decisions impact school food service within your school?
   1. In terms of the outer context surrounding the school, how would you say neighborhood factors influence school meal participation?
   2. What about district-level factors? How do decisions made at the district level impact food service?
3. What other factors outside the school setting do you feel are influential to implementing school meal programming?

**Wrap up:**

- That was the last question I have.
- What didn’t I ask about that you would like to share about school meals?
- Or anything you would like to ask me?
- Thank you so much. To thank you for your time, we will send you a gift card for $25 if you indicated in the survey that you would like to receive one.

**Food Service Focus Group Interview Guide**

**Introduction:**

Hi, my name is [insert name] and I'm from [insert organization]. Thank you for agreeing to talk with me today. I'd like to ask you a few questions about your thoughts on school meals. The information you share will be used for research purposes and to inform people working in your school and across the city of Philadelphia about how best to offer school meals. We'll combine all the results, and you and your school will not be identified with your responses. Your participation is voluntary. This should take about 30-45 minutes; you can stop anytime. There are no right or wrong answers. We just want to know what you think. If you have any questions, you can talk with the study's Principal Investigator, Gabriella McLoughlin. The contact information is in the consent form. I'm also planning to record our conversation. We'll delete the recording after we've written down your answers. And again, you won't be identified with your responses. Is it ok with you if we record this interview?

**Background/Context:**

1. First would you please say your name and position? (go around the group if it’s a focus group interview)
2. How long have you been this school’s food service director/staff? How have you found this role so far? (expand on things they like/dislike about their role)
3. Can you describe what a typical day in your role looks like?

[Construct - Implementation process]

1. What service model are you currently using for breakfast and lunch?
2. What are some challenges about preparing and delivering breakfast and lunch?
3. As far as you know, does your union have a policy or position about alternative breakfast and lunch service models?
4. If you had no constraints like staff, space or time, what would you change about the

content of breakfast and lunch and how they are delivered?

[Construct – Individuals domain]:

1. Can you tell me a little about your students and their lives outside of school? To your
2. knowledge, do families at this school struggle with food insecurity? Other health challenges?
3. What is your observation about students’ breakfast habits? What do you think students eat before they come to school, at home or on the way? What do you think about that?
4. What are your observations about students’ lunch habits? Do you see patterns or trends in participation in lunch? What are they and do you have any perceptions of influential factors to participation? (might not be needed if they talk about breakfast and lunch together)
5. How much do you feel students rely on school breakfast and lunch?
6. Do you observe students experiencing hunger, especially in the morning? What do you do?
7. What common comments do you hear about breakfast and lunch service from students?
8. How do you think parents view school breakfast and lunch service?
   1. To what degree are community members aware of/engaged in conversations about school meals?
9. What factors do you think are important to consider in promoting school meals to students/families?
   1. Demographics (i.e., race/ethnicity)
   2. Cultural factors (i.e., religion)
   3. Other?
10. To what degree do you think school meal programming is designed with these factors in mind currently?

[Construct - Inner Setting]:

1. Can you tell me a little about your school’s climate? How is attendance at your school?
2. How would you say the culture of school meals is within your school?
3. What’s the environment of the school cafeteria like? Does this differ throughout the day?
4. Who makes decisions about breakfast and lunch service at your school and how? (Both ordering the food itself and delivery model)
5. How do decisions about school meals typically get communicated between lunch service staff and teachers/administrators?
6. What training (if any) is provided to promote awareness/engagement around school meals?
7. To what degree do you think the school culture influences/is influenced by school food service?

[Construct - Outer Setting]:

1. Can you tell us about some of the city/district policies that may influence how school meals are served?
   1. What sort of an impact do they have?
2. What kinds of budgetary/financial decisions impact food service within your school?
   1. In terms of the outer context surrounding the school, how would you say neighborhood factors influence school meal participation?
   2. What about district-level factors? How do decisions made at the district level impact food service?
3. What other factors outside the school setting do you feel are influential to implementing school meal programming?

**Wrap up:**

- That was the last question I have.
- What didn’t I ask about that you would like to share about school meals?
- Or anything you would like to ask me?
- Thank you so much. To thank you for your time, we will send you a gift card for $25 if you indicated in the survey that you would like to receive one.
