# Supplementary material for: Understanding implementation determinants of universal school meals through an equity-driven mixed methods approach
Source: Implement Sci Commun. 2025 Apr 15;6:44. doi: 10.1186/s43058-025-00713-0 (PMC12001678; doi:10.1186/s43058-025-00713-0)
Supplement: Supplementary file 3 — Additional File 3: Observation Sheet [file 43058_2025_713_MOESM3_ESM.docx]

**School Meals Observation Sheet**

**School ID:**

**Date:**

**Time of observation start:**

**Time of observation ending:**

**Mealtime (breakfast or lunch):**

**If breakfast:** in the classroom hallway cafeteria other (describe)

**Grades served:**

**Menu for the day/options**

**Estimate of time spent eating (i.e., once most students have sat down to when they are finished/called up to leave)**

**Kitchen and cafeteria notes (i.e., food placement, overall environment, student entrance/transition, serving logistics)**

**Student participation/consumption/reactions/behaviors**

**Cleanup and student exit/transition**
