# Supplementary material for: Understanding implementation determinants of universal school meals through an equity-driven mixed methods approach
Source: Implement Sci Commun. 2025 Apr 15;6:44. doi: 10.1186/s43058-025-00713-0 (PMC12001678; doi:10.1186/s43058-025-00713-0)
Supplement: Supplementary file 4 — Additional File 4: Coding Consensus Document [file 43058_2025_713_MOESM4_ESM.docx]

**School Meals Needs Assessment Coding Consensus**

Guiding Framework: Consolidated Framework for Implementation Research V2

| **Domain and Construct** | **Coding Example/Source** | **Potential Misconceptions** |
| --- | --- | --- |
| **Ideas for the future** | Code examples here that relate to ideas or suggestions from participants for how school meals could be implemented better | Statements like “we need better food” don’t go here- instead in innovation design |
| **I. Innovation Characteristics** | **The degree to which…** |  |
| A. Innovation Source | The group that developed and/or visibly sponsored use of the innovation is reputable, credible, and/or trustable  Code: statements that indicate this policy is trustworthy, whether the state or federal government, and what this means for implementation.  e.g., “this policy comes from the government, so we want to make sure we implement it well” | Focus mainly on perceptions of the policy itself, and less on perceptions of the school district as that will be coded to outer setting factors. |
| B. Innovation Evidence Base | The innovation has robust evidence supporting its effectiveness  Code: perceptions of school meals and the benefit on students’ health, hunger, other school/student-level outcomes etc.  e.g., “having free school meals helps kids so much with hunger” or “it helps them throughout the school day” | Avoid putting responses that talk about why school meals do/don’t work for them or challenges. |
| C. Innovation Relative Advantage | The innovation is better than other available innovations or current practice  Code: perceptions related to school meals where participants compare it to other programs at school, in terms of its affect on student health or school climate.  e.g. “compared to (other program), school meals isn’t really that beneficial for our students” | Avoid coding issues of “priority” as this is in the Inner setting > “relative priority.  If comparing conditions between their school and another, this would probably go in > “available resources” |
| D. Innovation Adaptability | The innovation can be modified, tailored, or refined to fit local context or needs  Code: how adaptable is the school meals program? Is it flexible to the needs of the school context?  e.g., “we had to make changes to meal service, but that’s not too difficult since the policy is flexible”  “So I saw them throw like, over 100 milks away one time that we were down in the cafeteria, when the breakfasts were being dropped off. They had a huge biome, and they just threw them all away. I was like, Oh, my goodness. So that to me is like, Oh, that's a lot of money just being thrown away. I understand. Because it could go bad, because it's been out for so long. But it's like, there has to be a better way of knowing which classes want the milk on which days or if it's Cereal Day, like, this year, they've been better. So if there's five zeros, there's five milk, but sometimes I'll get 10 zeros in five minutes. And then I have to pour half the milk into one and then half into another” – shows this is not very adaptable | Ability to adapt, avoid coding the action of adapting as this is “adapting” |
| E. Innovation Trialability | The innovation can be tested or piloted on a small scale and undone  Code: elements of the policy such as menu changes, scheduling, or other aspects of the program.  e.g., “the district tried a menu change which went well” (we probably won’t be using this much since it’s a policy) |  |
| F. Innovation Complexity | Examples of how food service initially might have struggled but once they developed a plan they were able to implement. Difficulty with a complex program which impacted implementation.  Comments about how the program is not difficult also go here.  e.g., “it’s tricky because we have to order X foods and if we over order or under order it’s a challenge” | Avoid coding issues such as difficulty with getting buy-in from administration- code instead to engaging- leadership |
| G. Innovation Design | Code: Issues related to clarity of information, quality of resources, and training materials – especially for climate and food service staff.  Also code: food quality, preparation issues, portion size issues – perceptions of food quality, and menu decided issues  e.g., “the food isn’t good here, it was better a few years ago but now it’s cold and undercooked”  “no, only like the same things I said about like, we all get annoyed that the kids have to take all the components, and then they're throwing away food. (4513, Pos. 74)”  “They have to take the milk....there is a lot of waste sometimes” | Exclude statements regarding the presence or absence of materials (i.e., time, financial resources) and code to Available Resources.  If quote gives opinion with no context/example – code > innovation recipients |
| H. Innovation Cost | Code: Issues/perceptions of cost of the program. This might be most used for food service staff.  e.g., “we gotta stay under budget” (or other comments related to food costs).  Could also code issues related to how expensive it is to hire people and pay them to serve meals | If talking about not being able to afford certain foods or resources – > code to funding |
| **II. Outer Setting** |  |  |
| A. Critical Incidents | Large-scale and/or unanticipated events disrupt implementation and/or  delivery of the innovation  Code: how has the COVID-19 pandemic affected food service? Weather changes? Other events/issues affecting food service on a large scale  e.g., “since COVID we can no longer do X” |  |
| B. Local Attitudes | Sociocultural values (e.g., shared responsibility in helping recipients) and beliefs (e.g., convictions about the worthiness of recipients) encourage the  Outer Setting to support implementation and/or delivery of the innovation  Code: perceptions of school meals across the whole school community and alignment with culture/background of families/community members.  e.g., “a lot of kids don’t eat [food], it doesn’t really reflect the culture of the families we serve” (or comments from parents/students about disconnect between home and school in terms of what they eat at home differing from school) |  |
| C. Local Conditions | Economic, environmental, political, and/or technological conditions enable the Outer Setting to support implementation and/or delivery of the innovation  Code: factors within the local area/neighborhood affecting implementation of food service, socio-economic conditions of families around the school, etc. that might impact implementation.  e.g., “with the union negotiations we had to pivot food service” (include neighborhood safety here as well)  “My grandma cooks after she gets home from her shift at 10 pm” |  |
| D. Partnerships & Connections | The Inner Setting is networked with external entities, including referral networks, academic affiliations, and professional organization networks  Code: what partnerships exist? E.g., SNAP-Ed, community schools model, vetri foundation, other local partnerships to potentially support meals?  Also code any communication or lack thereof among school leaders and school district  e.g., “we don’t hear from the district at all” or “we have a great partnership with [organization]” | District communications/lack of communications go here |
| E. Policies & Laws | Legislation, regulations, professional group guidelines and recommendations, or accreditation standards support implementation and/or delivery of the innovation  Code: factors related to district, state, or federal policies affecting school meal implementation  e.g. “the USDA policy is limiting our ability to meet the students’ needs” or  “the district policy makes it so we can only choose from one food vendor” | Code issues related to how students must take a full meal into Innovation Design (we can change this later depending on what folks think) |
| F. Financing | Funding from external entities (e.g., grants, reimbursement) is available to implement and/or deliver the innovation  Code: financial issues affecting school meal implementation. i.e., is there enough funding? How does this affect meals implementation?  e.g., “the reimbursement model doesn’t allow us to serve healthy foods that are high quality, as food is very expensive now” | (reimbursement or grant related) |
| G. External Pressure | External pressures drive implementation and/or delivery of the innovation  Code: capture themes related to External Pressures that are not included in the subconstructs below) |  |
| 1. Societal Pressure | Mass media campaigns, advocacy groups, or social movements or protests  drive implementation and/or delivery of the innovation  Code: issues related to union negotiations, local political movements, etc. and how this might affect implementation.  e.g., “the recent strikes have caused disruption” |  |
| 2. Market Pressure | Competing with and/or imitating peer entities drives implementation and/or delivery of the innovation  Code: Any ongoing programs or initiatives that could pose a threat to school meal programs?  e.g., “we have vending machines at school so lots of the kids get junk food from there”  also include quotes about how corner/poppy stores are competing with breakfast uptake |  |
| 3. Performance Measurement Pressure | Quality or benchmarking metrics or established service goals drive implementation and/or delivery of the innovation  Code: pressure to increase participation in school meals, to be “under budget”, or other performance metrics.  Also code the issue of “increasing participation” from the school participant perspective and implications for this urgency.  “as long as we get participation up, that’s what the district cares about” |  |
| Procurement | Code issues/comments about obtaining food, deliveries, quality of food when it arrives/comes off the truck, and food supply  Also code issues related to food waste that can’t be coded elsewhere |  |
| **III. Inner Setting** | **The degree to which…** |  |
| A. Structural Characteristics | Code: Social structure- statements that refer to how the food service department or food service is peripheral or distal to the rest of their school, i.e., if they are in an isolated unit or if the school structure is unified. If decision making is centralized (i.e., “top-down approach”)  e.g., “there seems to be a disconnect between food service and the rest of the school” |  |
| 1. Physical Infrastructure | Layout and configuration of space and other tangible material features support functional performance of the Inner Setting  Code: Issues related to the layout of the cafeteria, how students line up for food, and other setup issues, also dirty/messy cafeteria  e.g., “the cafeteria is loud” or “because of how the tables are set up it makes it hard to spot students who don’t bring food” | Avoid coding issues related to “lack of space” as that will be in Available Resources. |
| 2. Information Technology Infrastructure | Technological systems for tele-communication, electronic documentation, and data storage, management, reporting, and analysis support functional performance of the Inner Setting  Code: issues with emails, online software or other systems that are reliant on technology, and how they impact meal implementation  e.g., “we have to put numbers of participation into the system but sometimes the system is down” |  |
| 3. Work Infrastructure | Organization of tasks and responsibilities within and between individuals and teams, and general staffing levels, support functional performance of the Inner Setting  Or, statements about the ability to “get things done” in their workplace culture setting.  Code: staffing issues, sharing of work duties/responsibilities  e.g., “They'll [other food service staff] do it. They'll do it with the fuss and all the talk behind it but it'll get done.”  “So I just send the check sheet the wall you have to cross it off now. I send that down the kids take the crate down on the trash. Okay, that's like one of their jobs today. But I like my grade partner and I will pick up the crates in the morning because it's too heavy for first graders even to I don't even think two kids could carry it's pretty heavy.” | Code issues with sharing of work duties/work allocations |
| B. Relational Communications | Code: Statements that refer to relationships and connections within the school setting i.e., **overall working relationships**  e.g. “So I do love that about them. They are definitely here and they go around and make sure that kids eat.” (1401, Pos. 144)  e.g., “And they're so picky about us like doing the sheet the right way and making sure the kids are taking all the components. But then sometimes the calendar doesn't even match. I'm like, Okay, well, they used to be picky about what we do (4513, Pos. 58)” | Don’t code “lack of buy-in” from other staff, and instead code these to engaging key stakeholders.  Code: Relationship dynamics within the school. |
| C. Communications | There are high quality formal and informal information sharing practices within and across Inner Setting boundaries (e.g., structural, professional)  Code: statements related to how school meals department communicates with other teams i.e., climate, admin, classroom, and communication within food service team  e.g., “we have regular **meetings** with climate and admin to talk about school meals or other issues coming up”  “we don’t communicate as a team” | Code specifically structured communications (meetings, emails) Ex: “Field trip form with food staff” |
| D. Culture | Code: When asked about their workplace culture, include statements about the degree to which the culture is a collaborative one or if there seems to be a culture of school meals. Negative examples could relate to how slowly things tend to move in school settings and a lack of clarity/opportunity for innovation.  e.g., “there’s a lot of stigma around school meals, and it’s a shame because it feels like this is something we can’t overcome”  e.g., “nobody eats the food, they just talk until lunch is over” | Comments specifically related to stigma as a problem should be coded here  Code social norms of students during mealtimes here  Social trends, not reactions🡪 innovation recipients  Also code any quotes related to culture of school meals |
| 1. Human Equality-Centeredness | There are shared values, beliefs, and norms about the inherent equal worth and value of all human beings  Code: statements related to perceived “equity” of implementation, whether they think all students are benefitting equally from school meals, etc.  e.g., “it’s a shame because when we get to the end of the lunch period, students don’t have the same choices as the kids who come in first, so they don’t get as good of an experience” |  |
| 2. Recipient-Centeredness | There are shared values, beliefs, and norms around caring, supporting, and addressing the needs and welfare of recipients  Code: how are students and families prioritized in school meal implementation? Issues related to their needs being met coded here.  e.g., “we care a lot about increasing participation but not a ton about what kids actually want to eat”  or “they took away XX from the menu without asking the kids even though it’s a top seller”  or “the kids don’t get a say in what we serve”  “I let kids eat during first period, they need to eat breakfast”  “They don't like those apple fritter circle things. Yeah, we and I they've been doing it for a few years now. And like we send so many of those back and when they put the extras out like before they're gonna expire. There's so many I wish that would be my only thing. Like I wish once the district realized there weren't a lot of kids It was like if they would substitute it for something else.” | If stigma mentioned code inner setting > culture and prioritize this code for how students input is valued in implementation  If talking about whether the district is asking for/seeking student input code to > assessing needs, innovation recipients |
| 3. Deliverer-Centeredness | There are shared values, beliefs, and norms around caring, supporting, and addressing the needs and welfare of deliverers  Code: perceptions of how food service staff are treated and their overall workplace satisfaction – how does this impact implementation?  e.g., “I feel like people listen to me and my ideas, and respect the food service team”  “it adds a lot of work to my plate and our needs aren’t really considered here” |  |
| 4. Learning-Centeredness | There are shared values, beliefs, and norms around psychological safety, continual improvement, and using data to inform practice  Code: statements indicating a care (or lack thereof) toward improving the school meals program and ensuring food service have what they need to implement properly.  e.g., “as a principal I want to improve the meals program, because I see the benefits on our students” | Try not to code issues such as evaluating, these go in reflecting & evaluating |
| E. Tension for Change | Code: Statements that allude to issues such as attendance, violence, lack of opportunity i.e., anything which states a NEED/deficit/problem that has to be addressed. Note: we might not be coding this as much since the policy has been in place for a long time and therefore the tension for change/need for something new wouldn’t really pertain to school meals.  e.g., “we needed to get attendance up at school”  or “we implemented breakfast in the classroom because kids weren’t eating” | Exclude comments linked to the benefits of school meals on student health outcomes.  Exclude other logistical changes made to school schedules such as recess etc. that isn’t too relevant to school meals, or some kind of “urgency” to improve student health outcomes |
| F. Compatibility | Code: perceptions related to how compatible the current school meals program is with the school culture/capacity. (we might not need to code this as much since it’s an ongoing policy and less of a new thing)  E.g. “I don’t think the school meals program is compatible with our current workflow and job demands” | Statements about the degree to which school meals is a priority for schools- code to culture if speaking more about general school culture or relative priority  If quote is talking about what students eat outside of school code to > local attitudes  If talking about if food is aligned with students needs/interests code to > recipient centeredness |
| G. Relative Priority | Code: The degree to which school meals are viewed as a priority in school operations, or “take a back seat” in terms of programming due to other pressures such as other after school programs/classroom instruction.  E.g. “I think that there are just too many other pressing issues schools are dealing with right now, food isn’t really seen as that important” | If school meals is not prioritized because it is not viewed as being compatible, code under compatibility. |
| H. Incentive Systems | Code: Statements related to internal incentives, payments or rewards for food service. Talk about lack of incentives also – as these could be interpreted as negative influences or capacity  E.g. “If I don’t do my job no one is really going to do anything about it, the kids just might not get all the components.” | If brought back to the need for the program, code under relative priority |
| I. Mission Alignment | Implementing and delivering the innovation is in line with the overarching commitment, purpose, or goals in the Inner Setting  Code: how do school meals align with the mission of the school system?  E.g. “this is what we do – as a school we make sure students are fed so they’re ready to learn” | Exclude statements that refer to the process or strategies used to implement.  Code:  -education outcomes and focus in terms of the whole school |
| J. Available Resources | Resources are available to implement and deliver the innovation  (Use this construct to capture themes related to Available Resources that are not  included in the subconstructs below)  E.g. “The **options** for us to order are very limited” or from student perspective regarding choice  Also “not enough food” is a limited resource, time, etc.  Also “inaccurate menu/not consistent options” coded here. | Save portion size complains for > innovation design but code running out of food/meals here |
| 1. Funding | Funding is available to implement and deliver the innovation  Code: issues related to funding – is the program well funded? Perceptions of whether the current model is supportive of implementation  E.g. “Yeah there are just some foods we can’t really afford to serve, they are just too costly” | If talking about how expensive the program is or how expensive it is to pay workers code to - > innovation cost |
| 1. Space | Physical space is available to implement and deliver the innovation  Code: factors such as cafeteria space, school crowding, kitchen space, etc.  E.g. “We actually had to create another lunchroom because we don’t have enough room for all the kids.” Or “the lunch line is so long” |  |
| 1. Materials & Equipment | Supplies are available to implement and deliver the innovation  Code: factors such as kitchen equipment, quantity of food available, and other material resources.  E.g. “Maybe if we had a full service kitchen, but without a proper kitchen we can only do the space meals.” |  |
| K. Access to Knowledge & Information | Guidance and/or training is accessible to implement and deliver or receive the innovation  Code: school staff (climate, food service) professional development, connection to field supervisors and other district support systems  E.g. “The only training we got was when we were hired, past that you just ask the manager how to do stuff.” | Avoid coding extracts which describe division of workload, tasks assigned to staff, etc. and code this in > Work Infrastructure |
| **IV. Individuals Domain** | **The roles and characteristics of individuals** |  |
| A. High-level Leaders | Individuals with a high level of authority, including key decision-makers,  executive leaders, or directors  Code: perceptions of whether/how school principals/administrators support school meals and how this impacts implementation  E.g. “The principal here is great at lunch, one time she even helped to serve when we were short staffed.” |  |
| B. Mid-level Leaders | Individuals with a moderate level of authority, including leaders supervised by a high-level leader and who supervise others  Code: perceptions of whether/how **school climate leaders/deans** support school meals and how this impacts implementation  E.g. “The climate manager here is really critical to helping us run lunch smoothly.” |  |
| C. Opinion Leaders | Individuals with informal influence on the attitudes and behaviors of others  Code: perceptions of whether/how school **classroom/other teachers and staff** support school meals and how this impacts implementation  E.g. “I have had teachers tell me that they eat the breakfast sometimes to show the kids it’s okay to eat.” |  |
| D. Implementation Facilitators | Individuals with subject matter expertise who assist, coach, or support implementation  Code: Support (or lack thereof) received from **field supervisors, community school coordinators,** and other facilitators (depending on each school) that support implementation  E.g. “I almost never hear from my field supervisor.” | If talking about communications and supports from district as a whole (not just field supervisors) code as > Partnerships & Connections |
| E. Implementation Leads | Individuals who lead efforts to implement the innovation  Code: perceptions (from others) related to the school **food service manager** and their capacity to fulfil the role  E.g. “Our manager this year is new so it’ll be interesting to see how the year goes” |  |
| F. Implementation Team Members | Individuals who collaborate with and support the Implementation Leads to implement the innovation, ideally including Innovation Deliverers and Recipients  Code: perceptions of if/how the **food service team** work together to deliver school meals  E.g. “I think we all work really well together, I always know if I’m not sure what to do I can ask anyone” |  |
| G. Other Implementation Support | Individuals who support the Implementation Leads and/or Implementation  Code: anyone else who isn’t assigned a formal role and their impact on implementation (e.g., parents, community members) |  |
| H. Innovation Deliverers | Individuals who are directly or indirectly delivering the innovation  Code: **climate staff** involvement in school meal implementation  E.g. “Climate walks around the cafeteria and make sure the kids don’t get too out of hand” |  |
| I. Innovation Recipients | Individuals who are directly or indirectly receiving the innovation  Code: insights related to how students and/or parents behave/react or are supporting/hindering implementation of school meals  E.g. “The student’s just don’t like the food, they say it’s nasty” (could also be from student perspective) | If talking about school demographics – code into outer setting > local conditions  If quote gives specific reactions related to /preparation code to > innovation design |
| **Characteristics Subdomain** | The degree to which: |  |
| 1. Need | The individual(s) has deficits related to survival, well-being, or personal fulfillment, which will be addressed by implementation and/or delivery of  the innovation  Code: statements highlighting food insecurity of students and families, and how the meals program alleviates hunger  E.g. “I do think that for a lot of these kids, this is the only meals they eat in a day.”  Also examples of how needs are being met |  |
| 1. Capability | Perceived ability (either within one individual or within the collective core team) regarding implementation. Negative examples could address lack of self or collective efficacy to implement the intervention.  Code: capacity of the school food service team/or school leaders to meet the needs of students and families with current school meals model  E.g. “It’s hard to do this job when you have to follow all these guidelines and keep all the kids happy” | If talk about engaging or trouble engaging key stake holders, code under leadership engagement |
| 1. Opportunity | The individual(s) has availability, scope, and power to fulfill role  Code: the degree to which food service/other school leaders and staff feel as if they have power and control to implement the school meals program and meet its potential. What support are they receiving?  E.g. “I just don’t think as a teacher I have any power over the school meals” |  |
| 1. Motivation | The individual(s) is committed to fulfilling role  Code: the food service team/ or anyone at school level are motivated (or perceived to be motivated) to do their best with implementing the school meals program.  E.g. “This is just something I feel really passionately about, I don’t think any kid should be going hungry” | Adapting because we care would be under motivation |
| **V. Implementation Process Domain** | **The degree to which school/district:** |  |
| A. Teaming | Code: Statements/data related to planning among school leaders and/or food service such as working together, meeting as a food service team, and other planning activities.  E.g. “We do meet with all of us to go over what is working well and what isn’t.” |  |
| B. Assessing Needs | Collect information about priorities, preferences, and needs of people  Use this construct to capture themes related to Assessing Needs that are not included in the subconstructs below |  |
| 1. Innovation Deliverers | Collect information about the priorities, preferences, and needs of deliverers to guide implementation and delivery of the innovation. Here is where we could tease out whether there is “shared” or “sole” leadership- i.e. how much collaboration existed within the core team and how much is it a one-person effort?  E.g. “They give us days where it is managers choice but other than that, they don’t ask food service what we think should be on the menu” |  |
| 1. Innovation Recipients | Collect information about the priorities, preferences, and needs of recipients to guide implementation and delivery of the innovation  Code: attempts to seek student/parent opinions/input on the school meals program  E.g. “I think the district needs to ask these kids what they want to see on the menu, they are the ones eating it everyday” | If talking about how students values/needs are prioritized without mentioning getting student input code > Recipient- Centeredness  Good spot for percentages, followed by an opinion about eating the meal. |
| C. Doing | Implement in small steps, tests, or cycles of change to trial and cumulatively optimize delivery of the innovation  Code: This might not be coded quite as much because the policy cannot be “trialed”. Instead, we code any insights related to trying new things with school meals, attempts to innovate, or other new ideas that come up  Again – this will likely not be coded much |  |
| D. Reflecting & Evaluating | Collect and discuss quantitative and qualitative information about the success of implementation. Note: Use this construct to capture themes related to Reflecting & Evaluating that are not included in the subconstructs below. | Exclude data which speaks to the (lack of) relation of school meals to ongoing goals and plans of the school- code to compatibility instead. Also, retrospective reflection and appraisal can go in the Doing construct. |
| 1. Implementation | Collect and discuss quantitative and qualitative information about the success of implementation  Code: any district- or school-level surveys or feedback mechanisms on school meal implementation (could be for students, parents, staff, etc.)  E.g., “I know they use nutrislice so that the kids can go on and rate the foods they like and don’t like.” |  |
| 2. Innovation | Collect and discuss quantitative and qualitative information about the success of the innovation  Code: insights on the school meals program itself and whether data are gathered  E.g. “We have done an internal audit to see what impacts participation” | Avoid coding extracts that talk about breakfast meal counts in the classroom and frustrations with that model – instead code that into work infrastructure or recipient centeredness |
| I. Adapting | Modify the innovation and/or the Inner Setting for optimal fit and integration into work processes.  Code: how (if at all) is the meals program being adapted and is this helping overall?  E.g. “We used to do breakfast in the cafeteria, but our new principal wanted to switch to classroom feeding.”  “What I do is what I do for my class, I have a basket and they put in there what they don't want, excuse me, and sometimes it'd be pretty full. So, if any of them do come arrive late, I'll make an exception because they're hungry you feed them and then what they would have left over I bring it in the lunch room and issue it to them because it is theirs I don't throw it away. Okay.” | Action of adapting.  If it’s an opinion on ability to adapt, code to Innovation Adaptability. |

| **Coding Valence and Strength** | **-2** | **-1** | **0** | **X** | **+1** | **+2** |
| --- | --- | --- | --- | --- | --- | --- |
|  | If school provided examples of how a specific facet hindered implementation or mentioned it repeatedly as a barrier | If mostly negative but some positive, try to code here to reflect the weight of the data. | General comments that relate to this construct but it’s difficult to tell if there is a positive or negative impact. | When there is discordance between two (or more) people on the same interview, and their points of view are about the same construct. Unless they come to an agreement or one’s point is addressing a more minor component. | If mostly positive but some negative, try to code here to reflect the weight of the data. i.e., “mostly positive” but some neutral or slightly negative. | If schools provided a concrete example of how this (construct) impacted implementation, or if they repeatedly mentioned how this positively influenced their implementation. |
