# Supplementary material for: Understanding implementation determinants of universal school meals through an equity-driven mixed methods approach
Source: Implement Sci Commun. 2025 Apr 15;6:44. doi: 10.1186/s43058-025-00713-0 (PMC12001678; doi:10.1186/s43058-025-00713-0)
Supplement: Supplementary file 5 — Additional File 5: Qualitative Coding Protocol [file 43058_2025_713_MOESM5_ESM.docx]

**School Meals Needs Assessment Qualitative Coding Protocol**

This protocol document serves as a step-by-step guide to coding the qualitative data (i.e., interviews, observations, field notes) in MAXQDA. Updates will be made iteratively as this is a working document. Team members can add updates and comments to this document through track changes, and the study lead will approve these changes as they arise.

Before you get started: Head to the MAXQDA TeamCloud Manual: <https://www.maxqda.com/help-mx24/teamcloud/welcome>

- Read through the manual – this should be your first stop if you have questions or want to explore certain features of the software.

**Step 1: Get all documents ready and open MAXWDA TeamCloud project**

- Open up MAXQDA and the TeamCloud project. Choose whether to work offline or online. Working online is better because you can just press “upload project for team lead” when finished, and this ensures a more seamless transition and collaboration (more below). If you want to work offline if you don’t have WIFI, you can export it as a local project and then re-upload it.


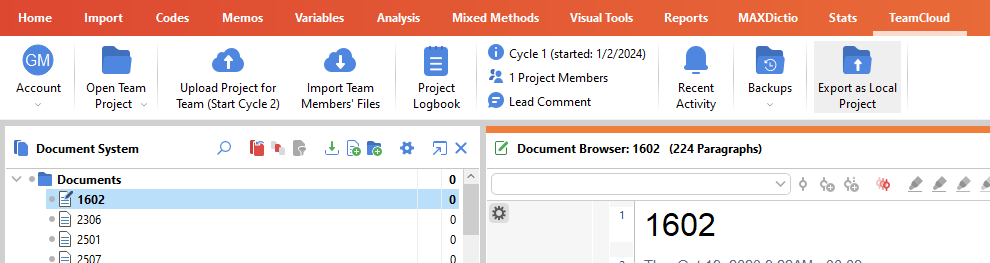


- Open up the [Interview Coding Workbook.xlsx](https://tuprd.sharepoint.com/:x:/r/sites/Community-DrivenImplementationLab/Shared%20Documents/General/Analysis/Qualitative%20Analysis/Interview%20Coding%20Workbook.xlsx?d=w7174a16bfb0141228def28dfc924e781&csf=1&web=1&e=FT3Tuh) that’s assigned to your name in the team folder
- Open up the [CDIL Coding Consensus Document.docx](https://tuprd.sharepoint.com/:w:/r/sites/Community-DrivenImplementationLab/Shared%20Documents/General/Analysis/Qualitative%20Analysis/CDIL%20Coding%20Consensus%20Document.docx?d=w6156b8ac217c40c991b56f996d761e24&csf=1&web=1&e=hnJqqK) – this is the guidebook that will help you distinguish what to code and where, as well as the rating rules for coding (at the bottom).

**Step 2: Select transcripts to code, and make sure code system and document browser are visible**

- In the Document System section (top right), go to “sets” and each school ID will show. From here, click the school you want to find and the transcript to code by the participant(s) ID number
- The three sections: documents, code system, and document browser should all be visible. If the “document browser”, “code system” or “document system” aren’t showing, go to “home” and select these. See in the screen shot below that these are all selected (in gray)
-
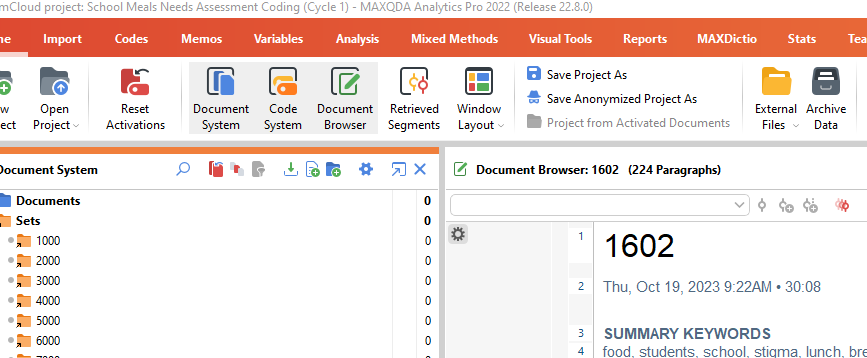
Give the transcript a quick read to spot any errors or missing bits of text.


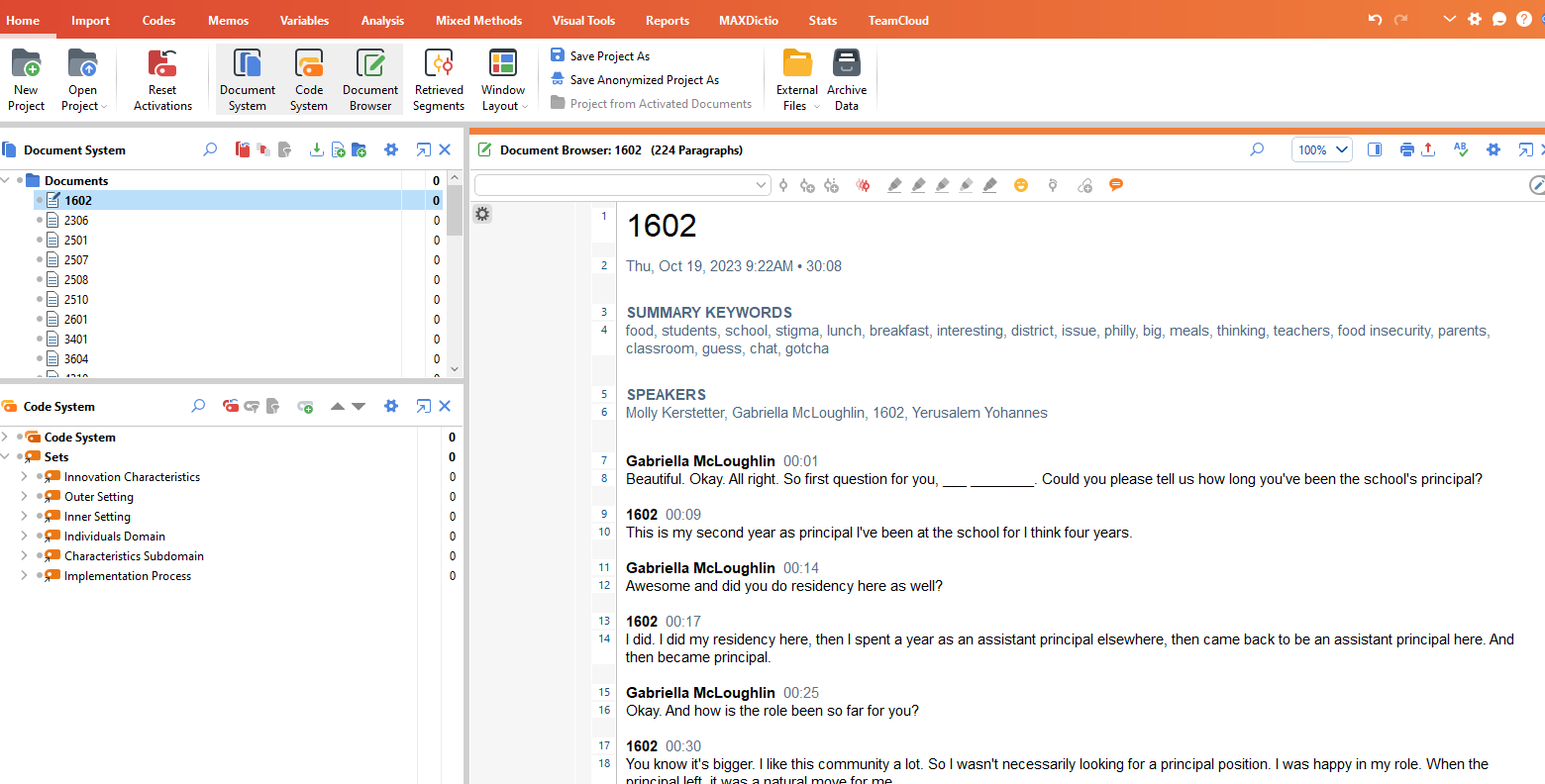


**Step 3: Coding with the CFIR Framework and adding numerical scores to data**

- When ready to code, start at the top of the transcript and read responses, searching for the first “codable piece of text”, or something that is important to document.
- Highlight the text that you want to code. Try to be as specific as possible here and make sure to highlight all relevant text, but not just a blanket paragraph if only one part of the paragraph is related. See below:


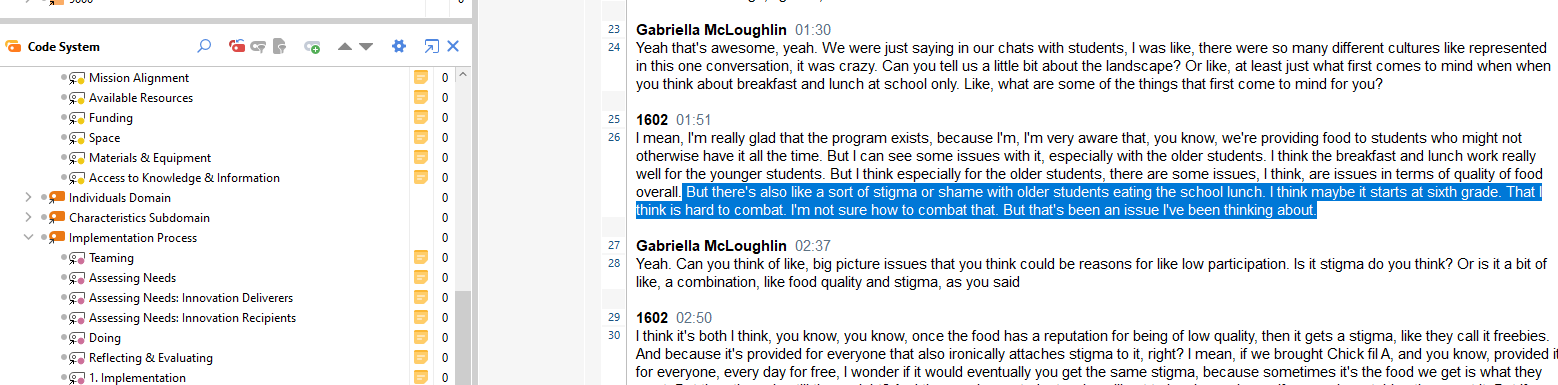


- **Coding option 1 – Preferred choice: “Click and Drag”** – This is more preferred because you can add comments easier and this helps with assigning the scores for each variable.
  - Drag the highlighted text and hover over the code you want it to belong to
  - Once the code shows up highlighted in blue, drop the segment in and see that the number on the right hand side increases by 1 (see below)


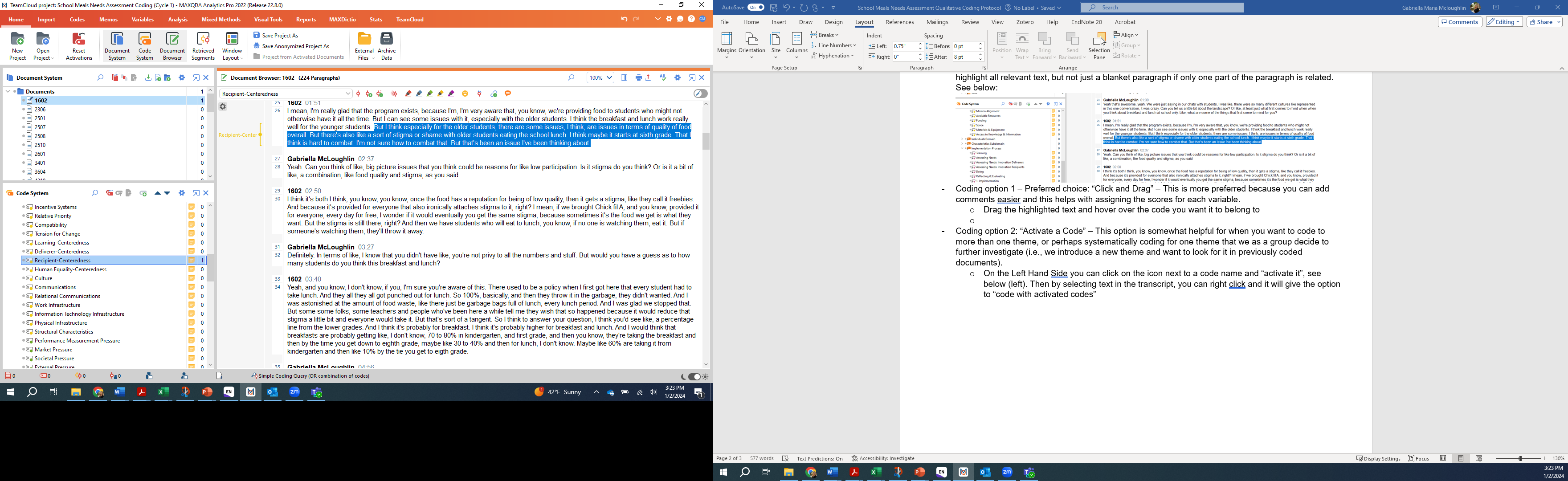


- **Coding option 2: “Activate a Code”** – This option is somewhat helpful for when you want to code to more than one theme, or perhaps systematically coding for one theme that we as a group decide to further investigate (i.e., we introduce a new theme and want to look for it in previously coded documents).
  - On the Left Hand Side you can click on the icon next to a code name and “activate it”, see below (left). Then by selecting text in the transcript, you can right click and it will give the option to “code with activated codes”


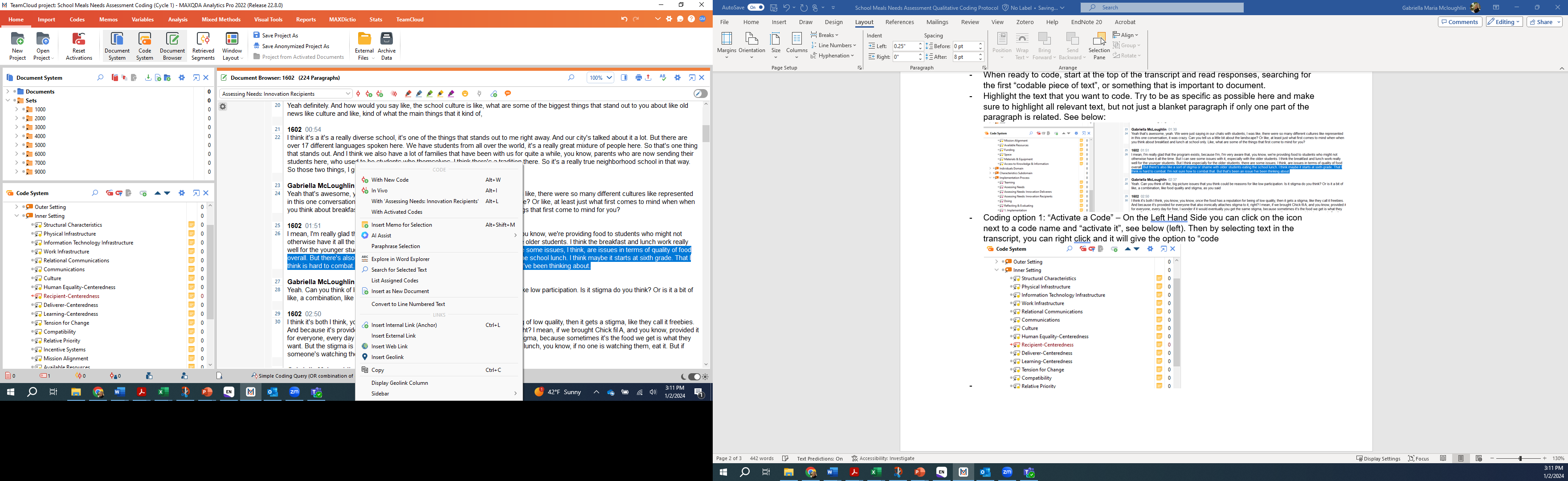

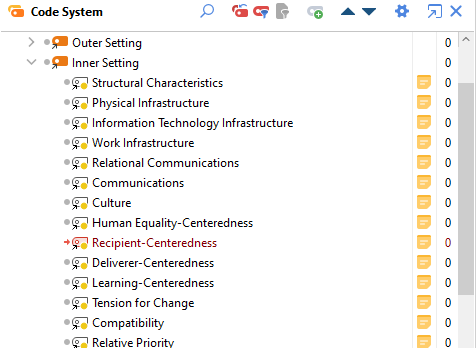


**Step 4: Adding comment and scores in coded segment**

- Once the segment has been coded, double click on the code and the below screen will appear (without comment added).
- We should also code interviewer quotes if necessary to understand extract – especially picking up on non-verbal cues
- Add your score (range from -2 to +2, including 0) to the comment and a justification (see below for example)
- For the weight score, we will leave this blank for now because you can’t add negative scores.


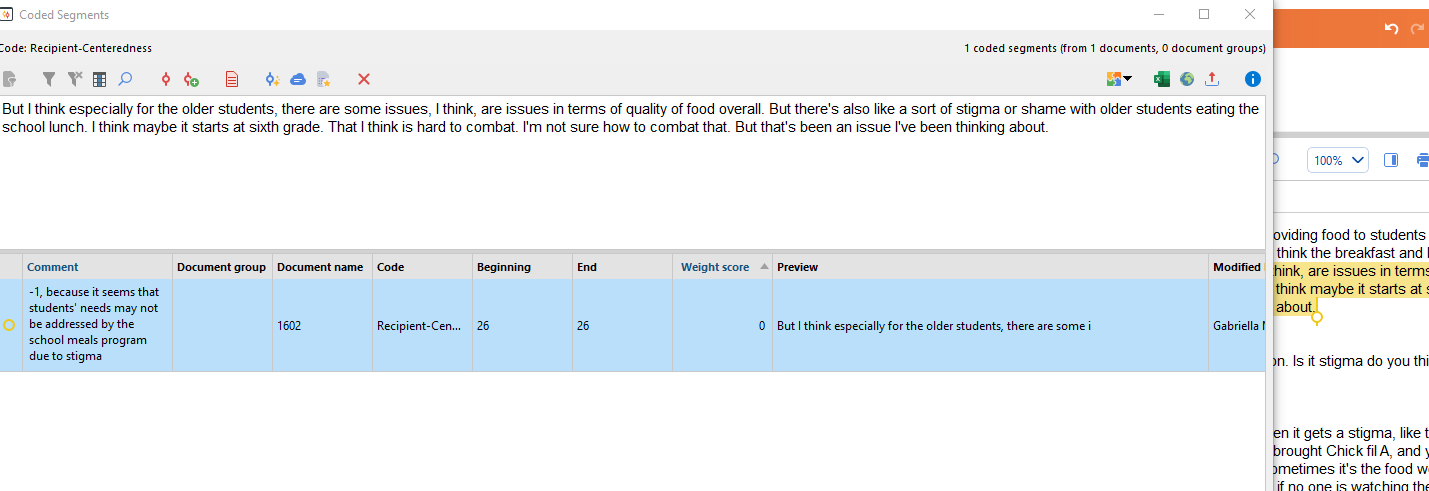


- Once comment has been added, you can hover over this coded piece in the document and all notes should be visible to make sure it’s been saved (see below)


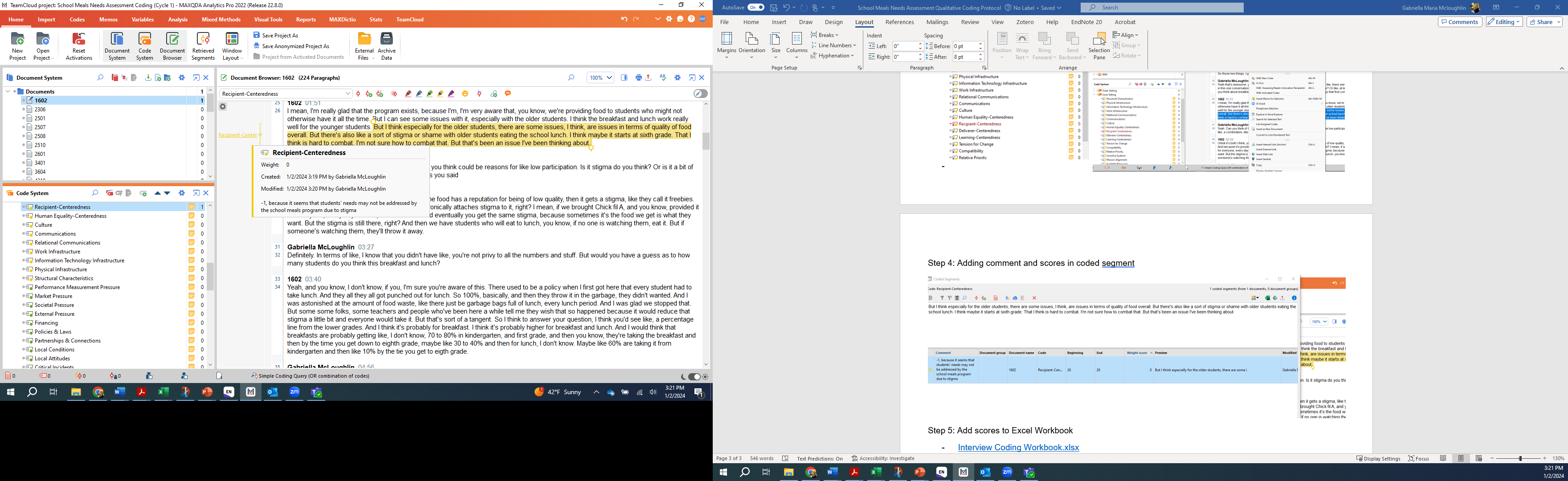


**Step 5: Add scores to Excel Workbook**

- Make sure the [Interview Coding Workbook.xlsx](https://tuprd.sharepoint.com/:x:/r/sites/Community-DrivenImplementationLab/Shared%20Documents/General/Analysis/Qualitative%20Analysis/Interview%20Coding%20Workbook.xlsx?d=w7174a16bfb0141228def28dfc924e781&csf=1&web=1&e=FT3Tuh) assigned to your name is open (since we will be coding as primary and secondary coders for the same transcripts and this avoids duplication)
- Add the transcript number you’re coding into Column A, then begin adding numerical scores into the same row corresponding to each domain and construct. See below:


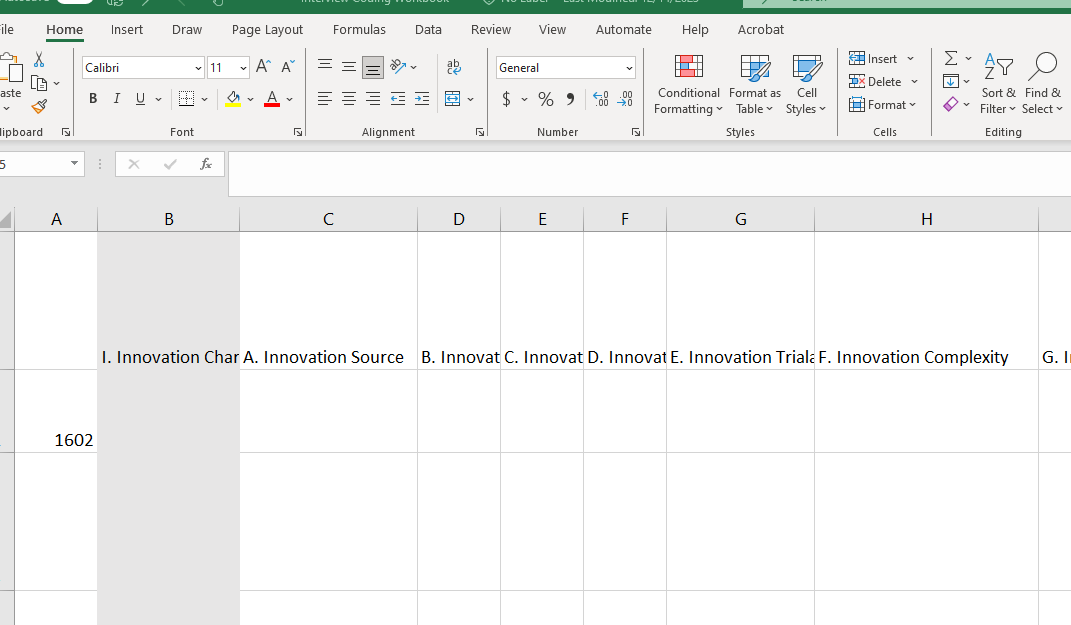


**Unsure about a certain piece of data?**

- Use the highlight feature and highlight text in yellow, followed by a memo to leave a note for the research team showing why you’re unsure about coding.

**Rinse and Repeat**

- Keep coding the whole transcript, and however many transcripts you’re able to complete. It’s best to code multiple transcripts in one “shift” if possible to keep the rhythm going.

**Save your work**

- Save your work on the Team Cloud, and when ready, upload to the online cloud. See screen shot below from the MAXQDA online manual:
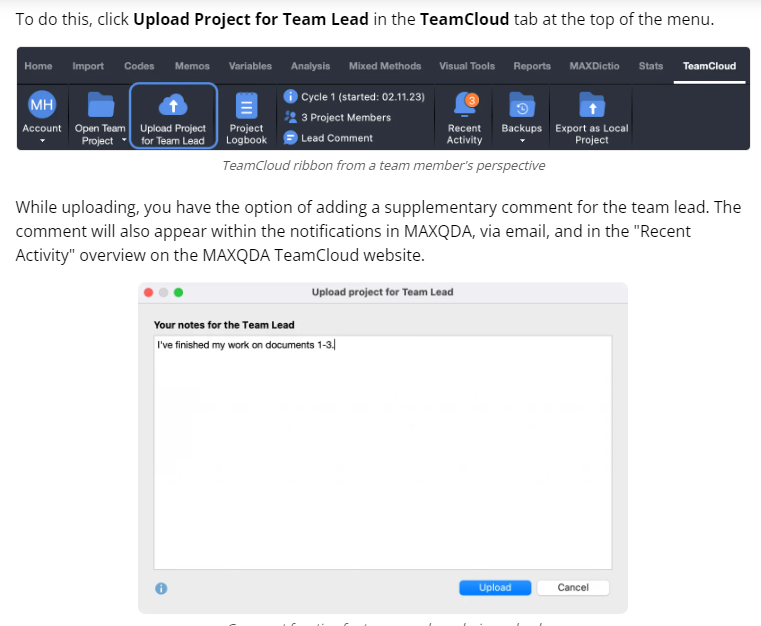


**Create Logbook Entry**

- Go to TeamCloud tab and “project logbook”

**
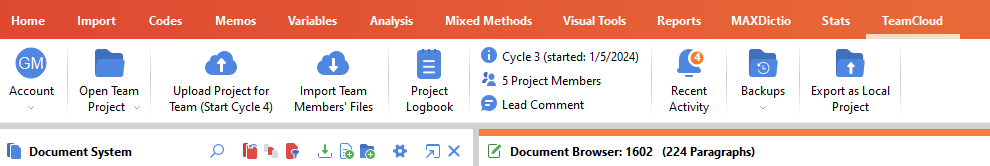
**

- Reflect on your coding for the week and discuss how it went. Feel free to use these prompts below:
  - What codes did you use the most?
  - What about the least?
  - What are some common themes you’re seeing?
  - Are there any codes we should discuss adding?
